# Selective steroidogenic cytochrome P450 haem iron ligation by steroid-derived isonitriles

**DOI:** 10.1038/s42004-023-00994-3

**Published:** 2023-09-02

**Authors:** Alaina M. Richard, Nathan R. Wong, Kurt Harris, Reethy Sundar, Emily E. Scott, Thomas C. Pochapsky

**Affiliations:** 1 Chemical Biology Program, University of Michigan, Ann Arbor, 48109 MI USA; 2 Dept. of Biochemistry, Brandeis University, 415 South St., Waltham, 02454-9110 MA USA; 3 Department of Medicinal Chemistry, University of Michigan, 428 Church St., Ann Arbor, 48109-1065 MI USA; 4 Departments of Pharmacology and Biological Chemistry, University of Michigan, Ann Arbor, 48109 MI USA; 5 Dept. of Chemistry and Rosenstiel Basic Medical Sciences Research Center, Brandeis University, 415 South St, Waltham, 02454-9110 MA USA

**Keywords:** Receptor pharmacology, X-ray crystallography, Solution-state NMR, Mechanism of action

## Abstract

Alkyl isonitriles, R—NC, have previously been shown to ligate the heme (haem) iron of cytochromes P450 in both accessible oxidation states (ferrous, Fe^2+^, and ferric, Fe^3+^). Herein, the preparation of four steroid-derived isonitriles and their interactions with several P450s, including the steroidogenic CYP17A1 and CYP106A2, as well as the more promiscuous drug metabolizers CYP3A4 and CYP2D6, is described. It was found that successful ligation of the heme iron by the isonitrile functionality for a given P450 depends on both the position and stereochemistry of the isonitrile on the steroid skeleton. Spectral studies indicate that isonitrile ligation of the ferric heme is stable upon reduction to the ferrous form, with reoxidation resulting in the original complex. A crystallographic structure of CYP17A1 with an isonitrile derived from pregnanalone further confirmed the interaction and identified the absolute stereochemistry of the bound species.

## all

The inhibition of cytochrome P450 (CYP) enzymes is central to many therapeutics, however, achieving selectivity across CYP enzymes remains challenging. Here, the authors develop isonitrile-harboring steroids as selective steroidogenic CYP heme iron ligands.

## Introduction

The inhibition of cytochrome P450 (CYP) enzymes plays an important role in many aspects of pharmacology and toxicology. For example, ritonavir and cobicistat are clinically significant CYP3A4 inhibitors that are part of antiviral “cocktail” treatments to slow degradation of other active components^1,2^. Examples include antiviral regimens for HIV and Pfizer’s FDA-approved oral SARS-CoV2 palliative Paxlovid^©^ (nirmatrelvir/ritonavir). P450 inhibition is also central to many antifungal therapeutics, with drugs such as fluconazole, itraconazole, miconazole, and voriconazole deriving their efficacy from their ability to strongly inhibit CYP51 (14α-sterol demethylase). This fungal P450 converts lanosterol obtained from the host organism to 14α-(desmethyl)lanosterol, an intermediate in the biosynthesis of ergosterol essential for fungal survival^3^.

Many pharmaceutically important P450 inhibitors make use of a planar nitrogen-containing moiety (e.g., imidazole, thiazole, triazole or pyridine) to ligate the heme (haem) iron of the target P450 enzyme via so-called “type II” binding. However, a major drawback to such inhibitors is their lack of selectivity across cytochrome P450 enzymes. For example, the type II antifungal P450 inhibitors fluconazole and voriconazole cross-react with human P450s, including CYP3A4, the P450 responsible for the metabolism of over 50% of all prescribed drugs^3,4^. Abiraterone, a pyridine-substituted pregnenolone-derived inhibitor of steroidogenic CYP17A1 used as a first-line treatment of prostate cancer, cross-reacts with human enzymes CYP11B1, CYP21A2, as well as CYP1A1, CYP2C9 and CYP3A4^5,6^. Given the degree of cross-reactivity of pharmaceutically important P450 inhibitors, there would appear to be a need for more selective inhibitors of these enzymes.

It has been known since the discovery of the first P450 enzyme that alkyl isonitriles (also known as isocyanides), R—NC, can also ligate the heme iron, and that such ligation strongly influences the heme Soret absorption band^7^. In the course of Omura and Sato’s isolation and characterization of microsomal cytochrome P450 enzymes, they found that treatment with ethyl isonitrile led to a Soret shift at 430 nm^7^.

A thorough investigation of the structural, spectroscopic and magnetic properties of *n*-butyl isonitrile complexes of cytochromes P450_cam_ and P450_nor_ by Lee et al. showed that isonitrile ligation is stable upon reduction of the heme iron, and that the reduced (Fe^2+^) P450-isonitrile complexes are diamagnetic (*S* = 0)^8^. This property could prove useful in our ongoing application of multidimensional nuclear magnetic resonance (NMR) to investigate the structure and dynamics of members of the P450 superfamily^9^. A primary difficulty for NMR-based investigations of P450 enzymes results from paramagnetism of the oxidized heme (*S* = 1/2 for low spin and *S* = 5/2 for high-spin ferric P450s). The enhanced nuclear spin relaxation due to unpaired electron spin density near the heme results in the short-circuiting of the coherence transfer pathways in the multidimensional NMR experiments typically used for sequential resonance assignments. Consequently, such experiments are often “blind” within a ~10 Å radius around the ferric heme iron (TCP, personal observation). This issue can be circumvented in P450 enzymes with small substrate binding pockets (e.g., P450_cam_) by heme iron reduction and axial ligation by carbon monoxide, generating a stable diamagnetic (*S* = 0) species. However, such complexes are insufficiently stable for long NMR experiments involving eukaryotic P450s with larger active sites, such as CYP17A1^10,11^. In the effort to generate ligands that might stabilize the reduced diamagnetic form of CYP17A1 for NMR studies, isonitrile derivatives of the commercially available steroids 5α-pregnanalone, trans-androsterone, and 5,6-dehydroepiandrosterone, were prepared. These compounds were also tested for their ability to ligate the heme iron of several other P450s to evaluate selectivity. These P450s include the bacterial steroid/terpenoid hydroxylase CYP106A2 and the human drug-metabolizing enzymes CYP3A4 and CYP2D6.

## Results

### Compound generation

**Fig. 1:**
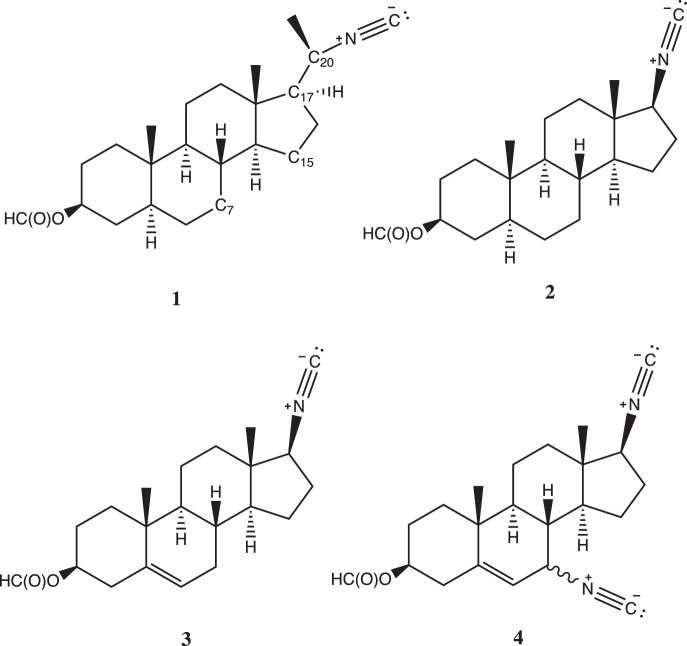
Structures of compounds **1–4**. Numbering for the C7, C15, C17, and C20 positions represented in compound **1**. Numbering follows conventions set for gonane and sterane-like steroids.

Compounds **1**–**4** (Fig. 1) were prepared using standard synthetic methods^12,13^, as described in Supplementary Information.

### Isonitrile binding and inhibition of CYP17A1

Absorbance of the Soret peak from the heme in cytochrome P450 enzymes is sensitive to iron interaction with ligands. Human CYP17A1 is isolated in the water-bound state (Soret λ_max_ = 417 nm) that blue-shifts upon substrate binding and red-shifts when coordinated with inhibitors. When human CYP17A1 was titrated with compounds **1**–**3**, only compound **1** induced the expected heme spectral shift from 417 nm (water-bound) to 430 nm consistent with formation of a ferric iron-isonitrile (Fe—C) dative bond. Compounds **2** and **3** were added to 25- and 20-fold molar excess, respectively, with compound **2** inducing a small shift to 420 nm, which is not consistent with isonitrile-heme coordination.

**Fig. 2:**
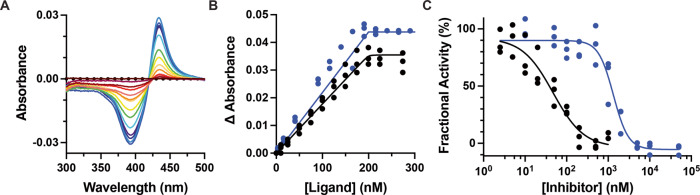
Binding and inhibition of CYP17A1 with compound **1** compared to abiraterone. **A** Titration of CYP17A1 with increasing concentrations (red→blue) of **1** yields difference spectra with increases at 430 nm, consistent with Fe—CN coordination. **B** Plots of the absorbance changes due to titration with **1** (blue) and abiraterone (black) reveal that both compounds bind very tightly. Protein concentration is 200 nM and the tight binding equation was used. **C** CYP17A1 progesterone hydroxylation is inhibited by both compounds, but more potently by abiraterone (black) than by **1** (blue). Both binding assays and inhibition assays were performed in triplicate and all points are shown, although some points overlap.

Compound **1** was originally prepared from a 2:1 mixture of C20 epimers of the formamide precursors (see Supplementary Information). The resulting 2:1 mixture of **1** was subsequently titrated into CYP17A1 and spectral changes observed in difference spectra, yielding a Soret peak shift to 430 nm, indicative of Fe—C bond formation. A single isomer of the formamide precursor of **1** was subsequently generated by recrystallization and the isonitrile generated from it bound to CYP17A1 with the same spectral shift (Fig. 2A) and with even higher affinity. This suggests that the stereochemistry of the compound near the heme-ligating isonitrile is important to optimize binding affinity.

Binding of **1** to CYP17A1 was compared to that of abiraterone, a CYP17A1 inhibitor used pharmaceutically as a first-line treatment of prostate cancer that forms a type II Fe—N interaction as evidenced by a shift of the Soret peak to 424 nm. Titration curves revealed both compounds are similarly tight-binding (Fig. 2B), with complete saturation by the time the ligand concentration equals protein concentration, suggesting a 1:1 stoichiometry for these ligands in the CYP17A1 active site. Even using the tight binding equation fitting this data estimates of 44 pM (95% CI, undefined to 3.8 nM) and 149 aM (95% CI, undefined to 1.7 nM) for abiraterone and **1**, respectively. However, comparison of inhibition by these two compounds yielded an IC_50_ for abiraterone 27-fold lower (47 nM) than for **1** (1300 nM) (Fig. 2C). This is may be due to steric constraints affecting the linearity of the Fe—C—N bond angle away from the likely optimum of 180° (vide infra). In contrast, the Fe-pyridinyl N bond formed with abiraterone is afforded more flexibility in terms of the rotation of the pyridine ring with respect to the remainder of the molecule. The slope of the inhibition curve for abiraterone is 1.1, consistent with the stoichiometry suggested by the binding assay and the structure identifying a single copy of abiraterone in the active site^14^. However, the slope of the inhibition curve for **1** is much steeper (2.4), which is not consistent with a 1:1 stoichiometry for ligand:P450.

### CYP17A1 structure confirms Fe—C isonitrile ligation and stereochemistry of 1

**Fig. 3:**
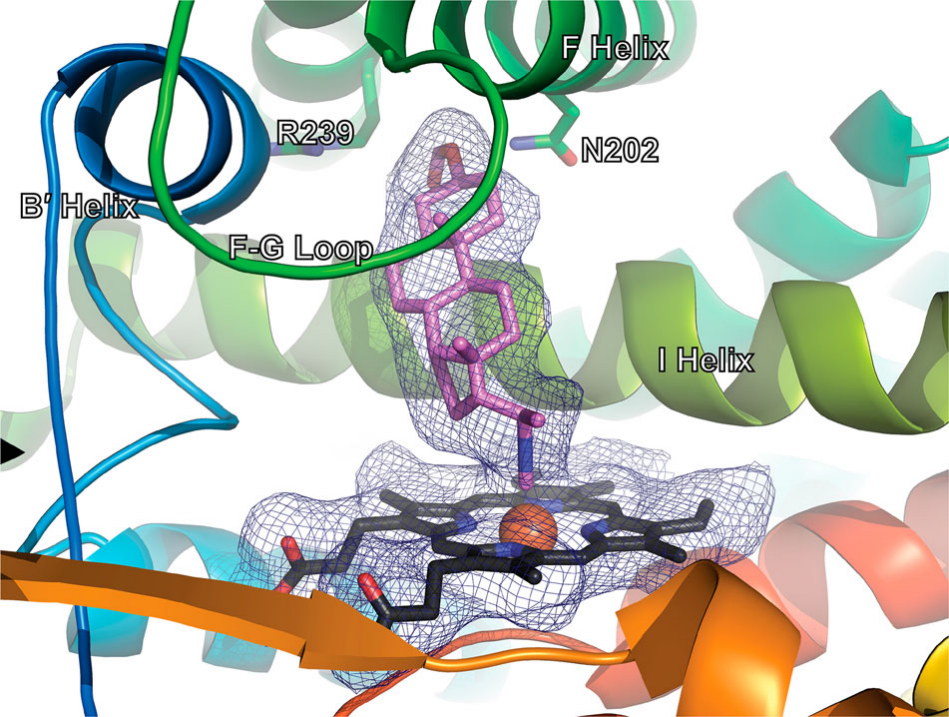
Crystallographic structure of CYP17A1 with the 20-(R)-**1** bound. The isonitrile substituent of 20-(R)-**1** coordinates the heme iron (PDB entry 8FDA). CYP17A1 in cartoons from blue N-terminus to red C-terminus. Heme in black sticks with orange sphere for Fe. Ligand in violet sticks. Electron density (2F_o_-F_c_ Polder map, 1 σ) in blue mesh for ligand and heme.

Co-crystallization of CYP17A1 with the tighter-binding epimer of **1** and subsequent X-ray diffraction analysis revealed that this is the 20-(R) epimer and confirmed formation of a bond between the carbon atom of the isonitrile functionality and the heme Fe (Fig. 3). The positioning of **1** in the active site is well-defined by the electron density, rising ~60° to the heme normal, orienting the C20 isonitrile group directly above the Fe. The C3 formyl ester substituent projects between N202, R239, and several waters, components that typically interact with both steroidal and nonsteroidal CYP17A1 ligands. The position of the heme-ligating isonitrile of **1** is similar to that of the pyridine nitrogen of abiraterone in those structures. The structure suggests a possible reason why compounds **2** and **3** with isonitrile functionality at C17 do not ligate the heme iron, while **1** with a C20 isonitrile does (*vide supra*). If the steroidal cores of **2** bind in the same position observed for **1**, the C17 isonitrile is directed in a different direction, away from the heme iron. In order for the C17 isonitrile of **2** to interact with the heme iron, the steroidal core would have to adjust about the long axis of the steroid, resulting in steric clashes, including those between the opposite C3 end of the molecule and I206 in the F helix. While we were unable to cleanly isolate the precursor for the S-(20) epimer of **1**, the importance of steroid position and orientation in the active site suggests that in order for a similar Fe—C bond to form with the S-(20) epimer of **1**, increased steric interactions between the C21 and C18 methyl groups would occur.

As is typical for CYP17A1 structures, two of the four monomers in the asymmetric unit have a slightly different conformation of the F/G and N-terminal regions. It has previously been reported that one of these conformations can accommodate a second lower-occupancy copy of the steroidal ligand distant from the active site^15,16^. Indeed, a second copy of **1** is bound in this peripheral site. Binding of this second copy of the ligand is also consistent with the steep slope observed in the inhibition experiment (Fig. 2c), suggesting a 2:1 stoichiometry, while the spectral shift binding assay only reports on the ligand molecule binding in the active site where it can cause a spin shift. However, in neither conformation does the binding of 20-(R)-**1** result in significant changes to the overall protein fold compared to structures with steroidal substrates or other steroidal inhibitors.

The crystallographic structure described here provides other insights into the stereochemical restrictions on effective Fe—C dative bond formation with isonitriles. Table 1 provides details of Fe—C—N bond angles as well as proximal Cys-thiolate—Fe bond and Fe—C bond distances from the four CYP17A1 molecules in the asymmetric unit. These are compared with the corresponding distances from the structures of *n*-butyl isonitrile complexes with P450cam and P450nor published by Lee et al. (PDB entries 1GEI, 1GEJ, 1GEK, and 1GEM)^8^. Note that the shorter Fe—C distances in subunits C and D relative to those in subunits A and B correspond with Fe—C—N bond angles approaching 180^°^, suggesting that the more linear Fe—C—N bond angle, the shorter (stronger) the Fe—C bond.

**Table 1:** Heme coordination geometry in P450-isonitrile complexes.

Complex	Cys-S—Fe distance (Å)	Fe—C (ligand) distance (Å)	Fe—C—N (ligand) angle (°)
P450_nor_(Fe^2+^)^a^	2.36	1.85	175
P450_nor_(Fe^3+^)^b^	2.33	1.86	171
P450_cam_(Fe^2+^)^c^	2.30	1.86	159
P450_cam_(Fe^3+^)^d^	2.24	1.82	149
17A1 chain A	2.70	2.64	152.33
17A1 chain B	2.34	2.78	149.93
17A1 chain C	2.69	2.13	171.06
17A1 chain D	2.69	2.13	163.58

PDB codes

^a^1GEI.

b1GEJ.

^c^1GEK.

^d^1GEM.

Complete description of the methodology and statistics of the structure determination are provided in Supplementary Information and Supplementary Table S1.

### Interactions of steroid-derived isonitriles with other key cytochrome P450 enzymes

**Fig. 4:**
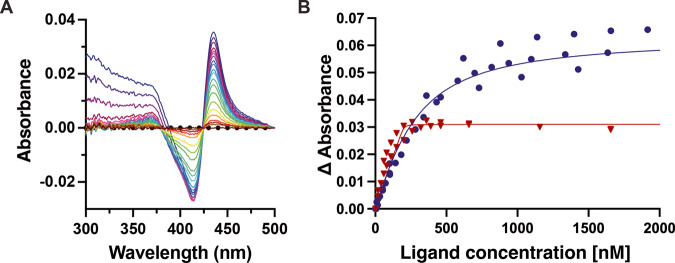
Binding of 20-(R)-**1** to human drug-metabolizing P450s. **A** Optical difference spectrum showing titration of CYP3A4 with of 20-(R)-**1** [0.01 (red) to 19.6 mM (violet)] also yields the difference spectrum peak at 435 nm indicative of Fe—CN bond formation. **B** A plot of the absorbance changes vs. ligand concentration reveals K_d_ values of approximately 163 nM for CYP3A4 (blue circles, *n* = 3, 95% CI 120–215 nM) and 0.49 nM for CYP2D6 (red triangles, *n* = 2, 95% CI undefined –6.4 nM), respectively. Protein concentration was 200 nM in a 5 cm cuvette. Data were fit to the tight-binding or quadratic equation.

To evaluate the selectivity of 20-(R)-**1**, this compound was also titrated against other cytochrome P450 enzymes, including the two most prevalent drug-metabolizing P450 enzymes in humans, CYP3A4 and CYP2D6. CYP3A4 is known to have a large and flexible active site^17^ so it is perhaps not surprising that titration of CYP3A4 with 20-(R)-**1** yields a Soret shift to 435 nM indicating Fe—CN—R bond formation (Fig. 4A). However, the dissociation constant (167 nM) for CYP3A4 is estimated at more than a billion times higher than for CYP17A1. Contribution of the isonitrile to affinity is apparent in that the parent steroid from which 20-(R)-**1** is derived, 3β-formyl-5α-pregnanalone, exhibits an ~20-fold higher K_d_ of ~3200 nM for CYP3A4, consistent with the Fe—C dative bond contributing ~6 kJ/mol to the free energy of complexation. Titration of CYP2D6 with 20-(R)-**1** also indicated the formation of the Fe—CN—R bond with a dissociation constant of 0.49 nM, which is 3 million times higher than CYP17A1 (Fig. 4B). Finally, 20-(R)-**1** was tested for its ability to inhibit CYP3A4-catalyzed nifedipine oxidation. In contrast to the 1.3 µM IC_50_ observed for CYP17A1, no inhibition of CYP3A4 was observed at concentrations up to 100 µM.

**Fig. 5:**
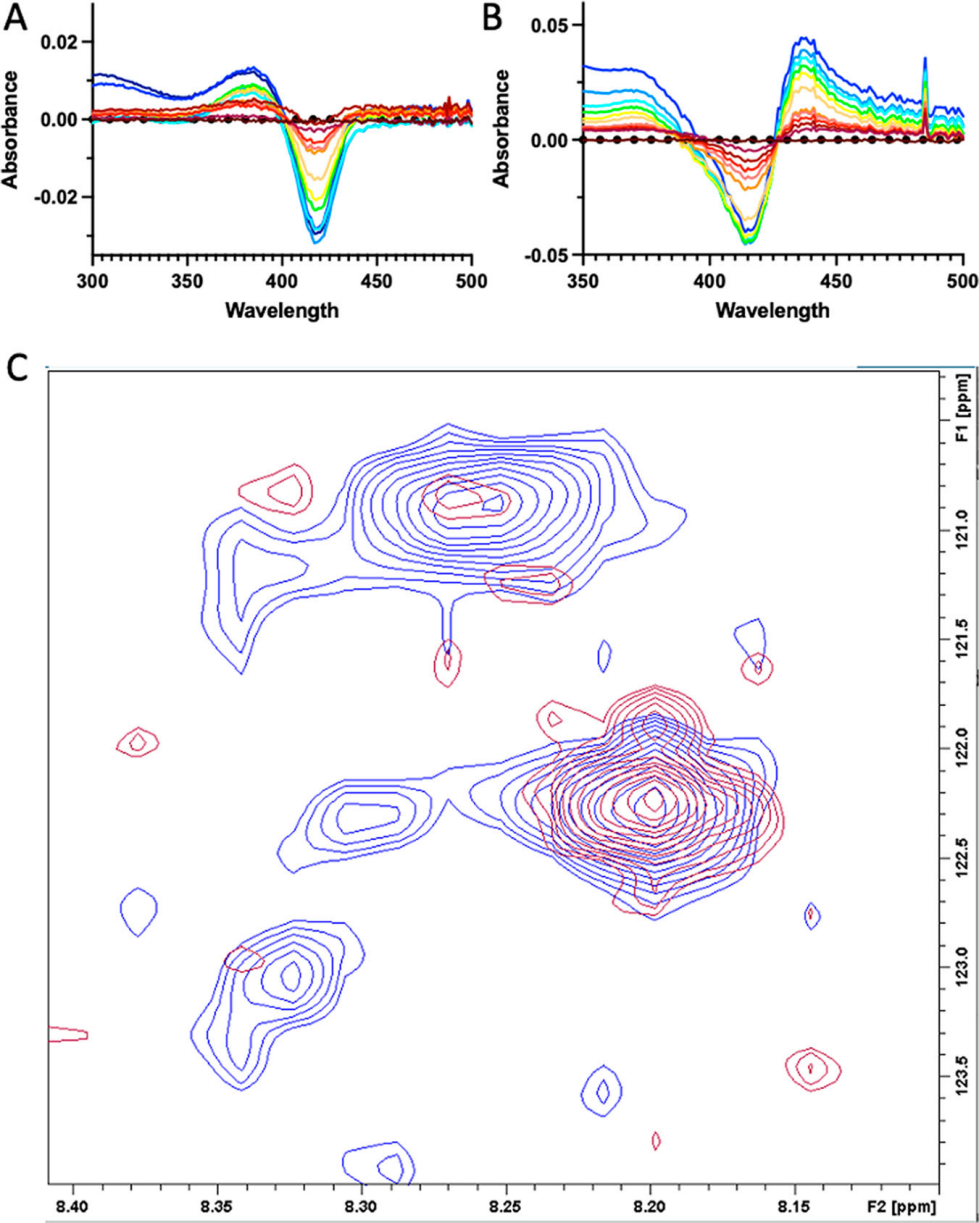
CYP106A2 interaction with steroidal isonitriles. **A** Absorbance difference spectra for titration of 1 µM CYP106A2 with **2** demonstrates blue shifted substrate-like “type I” binding (decrease at 422 nm and increase at 393 nm). Maximum concentration of **2** (light blue trace) is 20 µM. **B** Absorbance difference spectra for titration of 1 µM CYP106A2 with bis-isonitrile **4** demonstrates increasing absorbance at 435 nm consistent with formation of a heme Fe-isonitrile bond. Maximum concentration of **4** is ~10 µM (blue trace). **C** Overlay of 800 MHz ^1^H,^15^N-TROSY-HSQC spectra of ^15^N-Gly-labeled CYP106A2 with levopimaric acid p-benzoquinone Diels-Alder adduct bound after reduction with sodium dithionite and treatment with carbon monoxide (red), or with 7,17-(bis)isonitrile **4** (blue) after anaerobic reduction with sodium dithionite.

Unlike the human cytochrome P450 enzymes evaluated above, 20-(R)-**1** shows no evidence for formation of a Fe—C bonded complex with the bacterial steroidogenic CYP106A2. Rather, compound **2** results in a weak type I (substrate-like) blue shift of the Soret peak (Fig. 5A). While CYP17A1 hydroxylates steroids at C17, CYP106A2 hydroxylates steroids at C15 and C7^18^. Therefore a *bis*-isonitrile with a second isonitrile at C7 (**4**) was prepared. The crude product does form an iron-isonitrile bond upon binding to CYP106A2 (Fig. 5B). Thus, the proximity of the isonitrile functionality to the oxidized position(s) is likely important in determining whether an iron-isonitrile bond forms. As expected, the CYP106A2/**4** complex could be reduced under anaerobic conditions to form a stable diamagnetic complex suitable for NMR (see Fig. 5C). The appearance of a new NH correlation (120.8 ppm {^15^N} and 8.26 ppm {^1^H}) in the TROSY-HSQC spectrum of ^15^N-Gly labeled CYP106A2 after addition of **4** followed by reduction is strongly suggestive of the absence of paramagnetic broadening present in the original spectrum, indicating that the reduced CYP106A2/**4** complex is indeed diamagnetic^19^.

### Reduced cytochrome P450 interactions with steroidal isonitriles

**Fig. 6:**
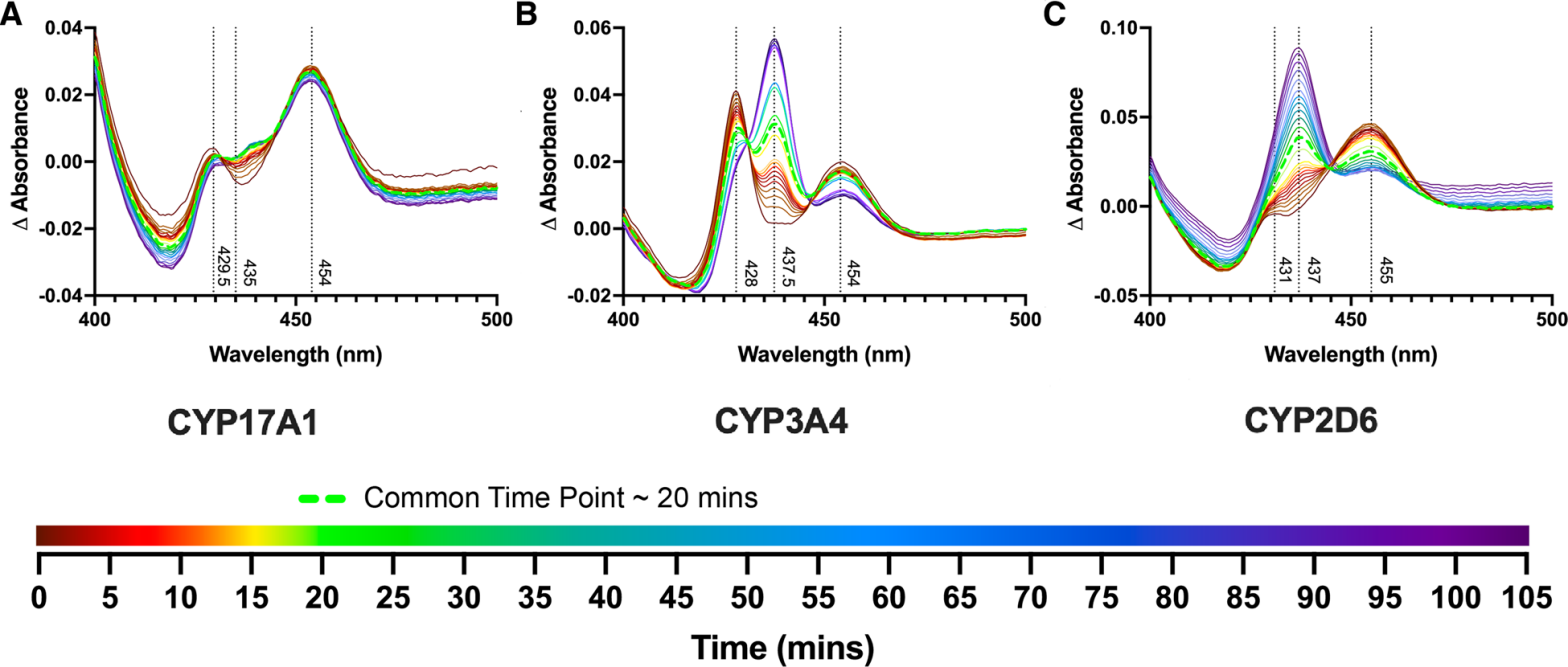
Reduced cytochrome P450 enzymes with 20-(R)-**1**. **A** CYP17A1/20-(R)-**1** complex after reduction by sodium dithionite yields a split Soret with maxima at 429.5 and 454 nm (brown initial spectrum), which exhibits little reoxidation even in air over 105 min. **B** CYP3A4/20-(R)-**1** complex after reduction by sodium dithionite also yields a split Soret with maxima at 428 and 454 nm (brown), which slowly re-oxidizes to the original 437.5  nm (purple, 103 min) in air over the same time period. **C** CYP2D6/20-(R)-**1** complex after reduction by sodium dithionite yields maxima at 431 nm and 455 nm (initial brown spectrum), which re-oxidizes in air to the original 437 nm over the same time period.

A significant advantage of isonitrile-based P450 ligands is their ability to bind both heme iron oxidation states. Current studies also suggested the ability of isonitriles to stabilize autooxidation. Reduction of the CYP17A1/20-(R)-**1** complex gives rise to a distinctive Soret doublet^8^ with absorbance at 430 and 454 nm (Fig. 6A, initial brown spectrum), with similar peaks for CYP3A4 and CYP2D6 (initial brown spectra, Fig. 6B, C, respectively). Evidence of the stability of the reduced isonitrile complex was investigated with all three P450 enzymes. Oxidized P450 enzymes saturated with 20-(R)-**1** were reduced with sodium dithionite and then allowed to re-oxidize in air (Fig. 6). For CYP3A4 and CYP2D6, reappearance of the oxidized Fe—CN ~435 nm peak and isosbestic points at 430 and 445 nm (CYP3A4) and 444 nm (CYP2D6) indicate a clean two-state conversion between the reduced and oxidized complexes with the isonitrile still bound, while CYP17A1/20-(R)-**1** was largely resistant to re-oxidation over the same time period. This result suggests that good complementarity between the CYP17A1 active site and the isonitrile ligand may not only promote binding in both oxidized and reduced states, but also stabilizes the heme iron towards oxidation.

## Conclusions

Initially, preparation of compounds **1–3** was directed solely at stabilizing a diamagnetic reduced form of CYP17A1 suitable for multidimensional NMR experiments. While **1** readily forms a tight-binding complex with CYP17A1, with apparent preference for the *R*-epimer, it binds less tightly to drug-metabolizing CYP3A4 and CYP2D6, and it does not cause spectral perturbation of CYP106A2, a bacterial steroidogenic P450. Conversely, compounds **2** and **3** did not give rise to spectral perturbations in CYP17A1, but appear to bind to CYP106A2 not by isonitrile ligation of the heme iron, but rather giving rise to a type I spectral shift (λ_max_ = 393 nm) typical of substrate binding. This suggests that both regio- and stereochemical constraints are important in formation of the Fe—C dative covalent bond and provides a rationale for the design of inhibitors for a given P450 based on this restriction. Indeed, compound **4** was prepared based on the known ability of CYP106A2 to hydroxylate steroids at C7-position, and it proved to bind with the shift expected for Fe—CN—R bond formation, in turn stabilizing the reduced form of that enzyme for NMR investigations.

Given their relatively straightforward preparation, the low toxicity of most isonitriles^20^ and their ability to bind to haemoproteins, it is perhaps surprising that steroid-derived isonitriles have not previously been investigated as pharmaceuticals^21^. Perhaps the inherent reactivity of the isonitrile functionality and instability under acidic conditions have discouraged such investigations in the past. However, herein it is established that compounds **1**–**4** are sufficiently stable in aqueous solution at physiological pH that standard binding assays are feasible, and appear to remain stably complexed to the P450 heme iron for multiple hours, during conversions between the ferric and ferrous oxidation states. Furthermore, recent advances in the delivery of enzymatically and/or chemically labile pharmaceuticals via encapsulation^22^ and targeted delivery^23^ suggests that compounds of this type may be feasibly adapted for medicinal purposes^24^.

## Methods

### Isonitrile syntheses

The synthesis of compound **1** is typical: 2 g of 3β-hydroxy-5α-pregnan-20-one (3.2 mmole) was added to 4 mL of 95% formic acid and 4.8 mL of formamide in a Pyrex test tube equipped with a magnetic stir bar. The test tube was stoppered with glass wool and heated to 165 ^°^C on an aluminum heating block with stirring and held at temperature for 3 h. After cooling, the two-phase mixture was mixed with sufficient benzene to dissolve the solid upper layer. The organic layer was filtered to remove unreacted starting material, then washed 2x with saturated NaHCO_3_ solution, dried over anhydrous Na_2_SO_4_, filtered, evaporated and recrystallized from benzene. Reversed phase HPLC of the first crop of recrystallized material (C_18_ column, acetonitrile/water gradient 20/80 − > 90/10, detection at 210 nm) showed evidence for several products, and ^1^H,^13^C NMR of the isolated fractions confirmed that R and S epimers of the formamide are present in ~2:1 proportion. The second crop of crystals from benzene, 0.78 g fine needles were obtained, m.p. 175–180 ^°^C, was determined by NMR to be essentially pure 3β-formyl-5α-pregnan-20-(R)-formamide, and was used for the isonitrile synthesis.

After drying over P_2_O_5_ in a vacuum dessicator, 0.265 mg (0.8 mmol) of recrystallized 3β-formyl-5α-pregnan-20-(R)-formamide was dissolved in 0.8 mL (3.2 mmole) of dry pyridine under N_2_ with stirring and cooled in an ice bath. 80 μL (0.8 mmol) of POCl_3_ was added slowly dropwise. After all of the POCl_3_ was added, the ice bath was removed, and the reaction allowed to proceed for ~2 h. The reaction mixture slowly darkened, and when no further color change was observed, the reaction was quenched with the addition of ice chips and 1 mL of saturated NaHCO_3_ solution. The reaction mixture was extracted with diethyl ether (5 mL × 3), the aqueous layer discarded and the organic layer filtered through anhydrous Na_2_SO_4_. Solvent was removed by a gentle stream of N_2_ without heating, and excess pyridine removed using a SpeedVac at room temperature. The resulting solid was examined by IR spectroscopy to confirm the presence of the isonitrile group, which exhibits a sharp absorption band at 2138 cm^-1^. Complete characterizations of intermediates and compounds **1–4** are provided in Supplementary Information, and relevant NMR spectra used to assign compounds are provided in Supplementary Data 1.

### Ligand binding assays

**(**Adapted from DeVore, et al.)^25^ Ligand interaction with the heme iron was evaluated by titrating ligands into a cuvette containing purified CYP17A1^14^, CYP2D6 L230D/L231R^26^, or CYP3A4^27^ and measured using a UV-visible scanning spectrophotometer. Enzyme was diluted to 0.2 µM in 100 mM potassium phosphate buffer (pH 7.4), 20% glycerol, and 100 mM sodium chloride and divided equally between two 5 mL cuvettes to evaluate high-affinity binding. Ligands dissolved in dimethylsulfoxide (DMSO) were added to the sample cuvette and an equal volume of DMSO to the reference cuvette. Difference spectra were recorded from 300 to 500 nm after equilibration of enzyme and ligand. Binding to the enzyme was measured as the difference in maximal and minimal absorbance: A_387_—A_419_ for pregnanolone and 3-formyl pregnanolone, and A_435_—A_393_ for compound **1** with CYP17A1; A_434_—A_414_ for compound **1** with CYP3A4 and CYP2D6. The dissociation constant and maximum spectral change were calculated using tight-binding or quadratic equation. All data fitting was accomplished using GraphPad Prism 9. Details of fitting are provided in Supplementary Information, and numerical data used to generate graphs in Figs. 2, 4 and 5 are provided in Supplementary Data 3 in Excel format.

### Reduction and reoxidation assays

Reduction of the **1**-bound P450 complex was observed by taking a baseline absorbance reading from 400 to 500 nm using a UV-visible scanning spectrophotometer, of a 1 cm quartz cuvette containing 1 µM P450, with compound **1** at a saturating concentration (i.e., ≥ concentration needed to reach ΔA_max_), in binding assay buffer (100 mM potassium phosphate buffer (pH 7.4), 20% glycerol, and 100 mM sodium chloride). A small quantity of sodium dithionite (spatula tip-full) was then added to the cuvette, and the spectra were recorded periodically over time to observe the formation of peaks indicating the reduction (~426 and 456 nm) and subsequent reoxidation (~435 nm) of the complex. The total time course for Fig. 6B was 103 min and 37 s. Numerical data used to generate graphs in Fig. 6 are provided in Supplementary Data 3 in Excel format.

*CYP17A1 Inhibition assay* (Adapted from DeVore & Scott)^14^. Metabolic activity of CYP17A1 was evaluated by measuring 17α-hydroxylation of progesterone as detected by HPLC with UV detection at 240 nm. A 1:4 ratio of CYP17A1 to recombinant NADPH-cytochrome P450 reductase was mixed and incubated on ice for 20 min. This mixture was added to buffer (50 mM Tris, pH 7.4, 5 mM MgCl_2_) containing 11.5 mM progesterone and either abiraterone (0–1 µM) or compound **1** (0–50 µM). Reaction vials were warmed to 37 °C for 3 min, then catalysis was initiated by adding NADPH to a final concentration of 1 mM. After 10 min, metabolism was quenched by adding 300 µL of 20% trichloroacetic acid and placed on ice. The reaction vials were centrifuged to pellet the precipitated protein, then the supernatant was injected onto a C18 column for HPLC identification of product. Metabolite elution was normalized to β-estradiol as an internal standard. A standard curve of known product concentrations was used to convert normalized area under curve to amount of product produced. A four-parameter variable-slope equation was used to fit the data and calculate IC_50_ values for inhibitors using GraphPad Prism 9. Details of the HPLC gradient used and data analysis are provided in Supplementary Information.

### CYP3A4 nifedipine metabolism assay

(Adapted from Bart & Scott)^28^. Metabolic activity of CYP3A4 was evaluated by measuring metabolism of nifedipine to dehydronifedipine (NFP), as detected by HPLC with UV detection at 254 nm. Reactions were carried out in amber microcentrifuge tubes (NFP is light sensitive) in a final volume of 150 μl. A 1:2 ratio of CYP3A4 to recombinant NADPH-cytochrome P450 reductase was mixed and incubated at room temperature for 20 min. This mixture was added to buffer (40 mM HEPES, 30 mM MgCl_2_, pH 7.4) containing 0.1 mM NFP and increasing concentrations of **1c** (0–100 µM). Reaction vials were warmed to 37 °C for 3 min, then catalysis was initiated by adding NADPH to a final concentration of 1 mM. After 20 min, metabolism was quenched by adding 50 µL acetonitrile and placed on ice. The reaction vials were centrifuged at 5000 × g for 5 min to pellet the precipitated protein, then the supernatant was injected onto a C18 column (Phenomenex, Luna, 50 × 4.6 mm) for HPLC. Separation on HPLC was obtained using a mobile phase of 45%/55% water/methanol for 40 min. A standard curve of known product concentrations was used to convert the area under the curve to the amount of product produced.

### Protein crystallization and structure determination

CYP17A1 was saturated with compound **1** and prepared for crystallization via hanging drop vapor diffusion. A protein solution of 30 mg/mL CYP17A1 in 50 mM Tris-HCl (pH 7.4), 20% glycerol, 500 mM NaCl, and 0.2% Emulgen 913 was equilibrated against 0.1 M Tris-HCl (pH 8.5), 0.25 M LiSO_4_, 30% PEG 3350, and 7% sucrose at 22 °C. Crystals appeared after 48 h. Crystals were cryoprotected in mother liquor supplemented with 24% glycerol and flash cooled in liquid nitrogen. Diffraction data was collected at 100 K at the Standford Synchrotron Radiation Laboratory beamline 12–2. Data were processed to 2.2 Angstroms using XDS^29^ and Scala^30^. The structure was solved by molecular replacement using PHASER^31^ with a search model based on CYP17A1 complexed with 3-keto-5a-abiraterone (PDB 6WW0)^15^. Iterative model building and structure refinement were accomplished using COOT^32^ and Phenix.refine^33^. Validation of this structure was performed in Phenix^33^. X-ray data statistics are provided in Table 2 and Supplementary Table S1. All figures were made using PyMOL^34^. Full coordinates are available as Supplemental Data 2.

**Table 2:** X-ray data collection, refinement, and validation statistics.

	CYP17A1 bound to 3β-formyl-5α-pregnanolone-(*R*)-20-isonitrile (compound 1)
Data collection	
Space group	P2_1_2_1_2_1_
Cell dimensions	
* A*, *b*, *c* (Å)	86.13, 152.27, 171.59
* α,β,γ* (°)	90.00, 90.00, 90.00
Resolution (Å)*	2.20
Redundancy*	13.4 (12.5)
*R*_pim_*	0.075 (1.453)
Mn(I/sd)*	6.8 (0.9)
CC ½*	0.997 (0.382)
Completeness* (%)	99.6 (99.1)
Total Reflections*	1,536,253 (206,362)
Unique Reflections*	114,766 (16,489)
Refinement	
Resolution (Å)	38.96–2.20
No. reflections	113,087
* R*_work_/*R*_free_	0.216/0.259
Number of non-hydrogen atoms / B factor	
Protein	14,871/55.8
Ligand	156/62.3
Heme	172/45.3
Solvent	268/49.6
R.M.S deviations	
Bond lengths (Å)	0.005
Bond angles (°)	0.73
Coordinate error (Maximum-likelihood) (Å)	0.30
Ramachandran plot: preferred/allowed/outliers (%)	96.7/3.2/0.11

*Highest-resolution shell shown in parentheses. Software used for structure refinement and visualization are referenced in Methods.

### Reporting summary

Further information on research design is available in the Nature Portfolio Reporting Summary linked to this article.

### Supplementary information

Supplemental Information

Description of Additional Supplementary Files

Supplementary Data 1

Supplementary Data 2

Supplementary Data 3

Reporting Summary

## Data Availability

Detailed synthetic methods used, compound characterizations, titration and inhibition experimental and analytical details, crystallization of CYP17A1-**1** complex and data collection, refinement and validation statistics are provided in Supplementary Information, which includes Supplementary Methods. NMR spectra used to characterize compounds **1–4** are provided as Supplementary Data 1. PDB format coordinates for the CYP17A1-**1** complex are available as Supplementary Data 2. Numerical sources for all graphs and titration plots can be found in Supplementary Data 3 in Excel format. The CYP17A1-**1** complex structure may be accessed as structure 8FDA with the following link: 10.2210/pdb8FDA/pdb.
