# Supplementary material for: Selective steroidogenic cytochrome P450 haem iron ligation by steroid-derived isonitriles
Source: Commun Chem. 2023 Sep 2;6:183. doi: 10.1038/s42004-023-00994-3 (PMC10475101; doi:10.1038/s42004-023-00994-3)
Supplement: Supplementary file 1 — Supplemental Information [file 42004_2023_994_MOESM1_ESM.pdf]

Supplementary information for:

Selective steroidogenic cytochrome P450 haem iron ligation using steroid-derived isonitriles.

Alaina M. Richard, Nathan R. Wong, Kurt Harris, Reethy Sundar, Emily E. Scott and Thomas C. Pochapsky

Supplementary Methods

S1) Synthesis and characterization of compounds 1-4 **pp. 2-6**

S2) Ligand binding and inhibition assays **pp. 6-7**

S3) Crystallization of 1-CYP17A1 complex and X-ray diffraction **p. 8**

S4) X-ray data collection, refinement, and validation statistics (Table S1) **p. 9**

S5) Supplementary references **p.10**

Supplementary Methods

S1) Synthesis and characterization of compounds 1-4

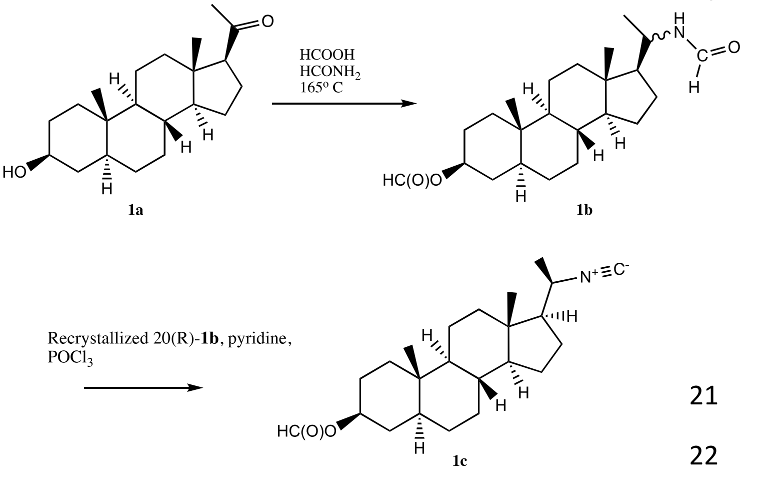

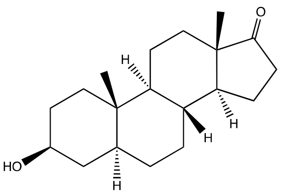

2a, epiandrostanone

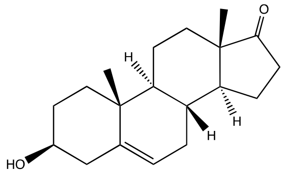

3a, 5,6-dehydroepiandrostanone

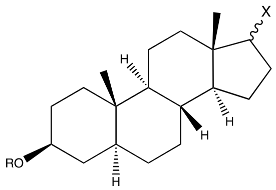

2b, R = H-(C=O), X= HN-COH
2c, R = H-(C=O), X = isonitrile

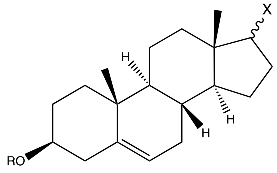

3b, R = H-(C=O)-, X= HN-COH
3c, R = H-(C=O), X = isonitrile

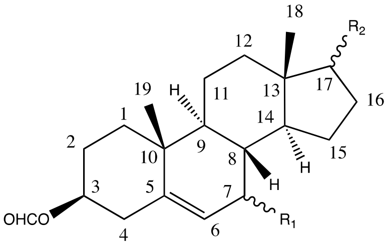

4a $R_1 = R_2$ = -NHCHO
4b $R_1 = R_2$ = -NC

3β-formyl-(R)-(20-amidoformyl)pregnane **1b.** 2g of 3β-hydroxy-5α-pregnan-20-one **1a** (3.2 mmole) (CAS 516-55-2, Oakwood Chemical, Estill SC) was added to 4 mL of 95% formic acid and 4.8 mL of formamide in a Pyrex test tube equipped with a magnetic stir bar. The test tube was stoppered with glass wool and heated to 165 °C on an aluminum heating block with stirring and held at temperature for 3 hours. After cooling, the two-phase mixture was mixed with sufficient benzene to dissolve the solid upper layer. The organic layer was filtered to remove unreacted **1a**, which is relatively insoluble in benzene, then washed 2x with saturated $NaHCO_3$ solution, dried over anhydrous Na_2SO_4, filtered, evaporated and recrystallized from benzene. Reversed phase HPLC of the first crop of recrystallized material (C_{18} column, acetonitrile/water gradient 20/80 -> 90/10, detection at 210 nm) showed evidence for several products, and ^1H,^{13}C NMR of the isolated fractions confirmed that R and S epimers of the formamide are present in ~2:1 proportion. Furthermore, two conformational isomers were observed at slow exchange om the ^1H chemical shift time scale for both epimers, presumably due to slow interconversion of the *cis* and *trans* NH-CHO forms (only the more upfield

^{1}H shifts for the two forms are reported below). Finally, the 3β-hydroxy group was esterified by formic acid in ~90% of the first recrystallization, based on NMR signal intensities.

The second crop of crystals from benzene, 0.78 g fine needles were obtained, m.p. 175-180 °C, was determined by NMR to be essentially pure 20-(R)-**1b**, and was used for the isonitrile synthesis.

***)^{1}H NMR** (d_6-benzene): H1 (0.74, 1.50); H2 (0.72,1.50); H3 (4.83); H4 (1.31,1.55); H5 (0.86); H6, 1.06; H7, 1.31, 1.54; H8, (R_{20}, 1.13; S_{20} 1.80); H9 (0.42); H11 ($S_{20,}$ 1.10, 1.35; $R_{20}\alpha$, 1.44; $R_{20}\beta$, 0.87); H12, 1.76, 1.47; H14, 0.77; H15 ($S_{20}\alpha$ 1.50, $S_{20}\beta$, 2.34; $R_{20}\alpha$, 1.31; $R_{20}\beta$, 1.11); H16 ($S_{20}\alpha$ 1.03, $S_{20}\beta$, 1.59; $R_{20}\alpha$, 1.72; $R_{20}\beta$, 0.82); H17 (S_{20}, 0.98 R_{20}, 0.87); H18 (S_{20}, 0.56; R_{20}, 0.63); H19 (0.59); H20 (S_{20}, 4.02; R_{20}, 4.06); H21 (S_{20}, 0.97; R_{20}, 0.85), formamide NH (S_{20}, 5.28, exch. 4.04; R_{20}, 5.86, exch. 4.19); formamide H(CO) (S_{20}, 7.59; R_{20}, 7.74); formate H(CO) (7.74)

^{13}C NMR (d_6-benzene): C1, 33.3; C2, 38.0; C3, 74.5; C4, 35.5; C5, 45.0; C6, 29.8; C7, 35.5; C8, 36.6; C9, 55.6; C10, 37.4; C11, 25.2; C12, 29.0; C13 (R_{20}, 42.9; $S_{20,}$45.9); C14, 57.6; C15, ($S_{20,}$ 21.6; $R_{20,}$ 22.5); C16 (R_{20}, 40.9; $S_{20,}$ 40.0); C17 (R_{20}, 57.7; $S_{20,}$ 56.4); C18 (R_{20}, 13.8; $S_{20,}$ 12.6); C19, 13.3; C20 (R, 46.4; S, 50.4); C21 (R_{20}, 22.7; S_{20}, 23.3); formamide carbonyl (R_{20}, 160.4; S_{20}, 163.7); formate carbonyl (161.3).

^{15}N NMR (in d_6-benzene): formamide R_{20}, 136.1; S_{20}, 102.6.

HRMS (1b): calculated for $C_{23}H_{38}NO_3$ (M+1), 376.2852, observed, 376.2835

Peaks eluted from C18 reverse phase HPLC (acetonitrile/water gradient), 72% ACN and 73% ACN.

3β-formyl-(R)-(20-isonitrilo)pregnane **1c**. (Identified as compound **1** in the Communication) After drying over P_2O_5 in a vacuum dessicator, 0.265 mg (0.8 mmol) of recrystallized 20-(R)-**1b** was dissolved in 0.8 mL (3.2 mmole) of dry pyridine under N_2 with stirring and cooled in an ice bath. 80 µL (0.8 mmol) of $POCl_3$ (Sigma) was added slowly dropwise. After all of the $POCl_3$ was added, the ice bath was removed, and the reaction allowed to proceed for ~2 h. The reaction mixture slowly darkened, and when no further color change was observed, the reaction was quenched with the addition of ice chips and 1 mL of saturated $NaHCO_3$ solution. The reaction mixture was extracted with diethyl ether (5 mL x3), the aqueous layer discarded and the organic layer filtered through anhydrous Na_2SO_4. Solvent was removed by a gentle stream of N_2 without heating, and excess pyridine removed using a SpeedVac. The resulting solid was examined by IR spectroscopy to confirm the presence of the isonitrile group, which exhibits a sharp absorption band at 2138 cm^{-1}.

^{1}H NMR 1c (d_6-benzene): H1, 0.70, 1.43; H2, 1.41,1.73; H3, 4.79; H4, 1.27,1.51; H5, 0.85; H6, 1.01,1.05; H7, 0.69, 1.45; H8, 1.01; H9, 0.32, 0.39; H11, 1.06, 1.30; H12, 0.76, 1.52; H14 ($R_{20,}$ 0.68); H15 ($R_{20}\alpha$, 1.45; $R_{20}\beta$, 1.84); H16 ($R_{20}\alpha$, 1.42; $R_{20}\beta$, 0.72); H17 (R_{20}, 1.12); H18 (R_{20}, 0.34); H19, 0.56; H20 (R_{20}, 3.11); H21 (R_{20}, 0.75).

^{13}C NMR 1c (d_6-benzene): C1, 37.1; C2, 28.1; C3, 73.2; C4, 34.7; C5, 44.9; C6, 28.9; C7, 32.5; C8, 35.4; C9, 54.5; C10, 36.9; C11, 21.4; C12, 32.7; C13 (R_{20}, 43.0; $S_{20,}$ 42.3); C14 ($R_{20,}$ 56.3); C15 ($R_{20,}$ 26.9); C16 ($R_{20,}$ 39.1); C17 ($R_{20,}$ 55.8); C18 ($R_{20,}$ 12.5); C19, 12.4; C20 (52.4); C21 (R_{20}, 22.4); isonitrile C (R_{20}, 158.1 (broad)).

HRMS (1c): calculated for $C_{22}H_{35}NO_2$ (M+1), 358.2747, observed, 358.2741

1c was eluted from C18 reverse phase HPLC (acetonitrile/water gradient), 95% ACN.

3β-formyl-(R,S)-(17-amidoformyl)androstane **<u>2b</u>.** 1g of 3β-hydroxy-5α-androstan-17-one **1** (2.9 mmole) (CAS 481-29-8, Sigma) was added to 2 mL of 95% formic acid and 2.4 mL of formamide in a Pyrex test tube equipped with a magnetic stir bar. The test tube was stoppered with glass wool and heated to 175 ^{o}C on an aluminum heating block with stirring and held at temperature for 4 hours. After cooling, the solid mass was extracted with CH_2Cl_2. After removal of solvent from the extract, the product was recrystallized from a minimal amount of ethanol and CH_2Cl_2 by slow evaporation. Based on relative NMR signal intensities, the recrystallized product is approximately 95:5 S_{17}:R_{17} epimers.

^{1}H NMR <u>2b</u> (d$_6$-benzene): H1, 1.38, 1.59; H2, 1.52,1.80; H3, 4.77; H4 1.64,1.69; H5, 1.12; H6, 1.39,1.77; H7, 0.86, 1.63; H8, 1.32; H9, 0.62; H11, 1.20, 1.51; H12, 0.95, 1.55; H14, (R_{17}, 1.03; S_{17} 0.93) H15 (R_{17}, 1.15, 1.67; S_{17}, 1.17,1.61); H16 (R_{17}, 2.21,1.29; S_{17}, 1.96,1.22); H17 (R_{17}, 3.31; S_{17}, 3.10); H18 (R_{17}, 0.71; S_{17}, 0.64); H19 0.78; formamide 17-HN (R_{17}, 7.09; S_{17}, 7.15); formamide 17-HCO (R_{17}, 7.86; S_{17}, 7.93); 3-HCO, 7.96.

^{13}C NMR <u>2b</u> (d$_6$-benzene): C1, 33.9; C2, 27.3; C3, 73.5; C4, 36.7; C5, 44.5; C6, 31.2; C7, 31.5; C8, 35.5; C9, 54.1; C10, 35.5; C11, 20.5; C12, 36.5; C13 (R_{17}, 44.8; S_{17}, 42.9); C14 (R_{17}, 50.0; S_{17}, 52.4); C15 (R_{17}, 24.6; S_{17}, 23.4); C16 (R_{17}, 29.8; S_{17}, 28.3); C17 (R_{17}, 61.6; S_{17}, 63.0); C18 (R_{17}, 18.1; S_{17}, 11.6); C19, 12.1; formamide carbonyl (R_{17}, 165.2; S_{17}, 165.1); 3-CHO, 160.9.

^{15}N NMR <u>2b</u> (d$_6$-benzene): formamide S_{17}, 115.6; R_{17}, 121.7.

HRMS (2b): calculated for $C_{21}H_{34}NO_3$ (M+1), 348.2539, observed, 348.2526

<u>2b</u> eluted from C18 reverse phase HPLC (acetonitrile/water gradient), 70% ACN (R_{17}) and 72% ACN (S_{17}).

3β-formyl-(R,S)-(17-amidoformyl)androst-5,6-ene **<u>3b</u>.** 1g of 3β-hydroxy-5α-androst-5,6-ene-17-one **3a** (2.9 mmole, CAS 481-29-8, Sigma) was added to 2 mL of 95% formic acid and 2.4 mL of formamide in a Pyrex test tube equipped with a magnetic stir bar. The test tube was stoppered with glass wool and heated to 175 ^{o}C on an aluminum heating block with stirring and held at temperature for 6 hours. After cooling, the solid mass was extracted with CH_2Cl_2. The product was recrystallized from a minimal amount of hexanes and CH_2Cl_2 by slow evaporation, m.p. 255 ^{o}C, By NMR, product formamide was determined to be >9:1 S_{17} :R_{17}.

^{1}H NMR <u>3b</u> (d-chloroform): H1, 1.16, 1.89; H2 1.65, 1.90; H3, 4.73; H4, 2.35 ; H6, 5.40; H7, 1.58, 2.01; H8, 1.32; H9, 1.16; H11, 1.35, 1.63; H12, 1.08,1.75; H14, (R_{17}, 1.05; S_{17} 1.13); H15 (R_{17}, 1.44, 1.61; S_{17}, 1.40, 1.58); H16 (R_{17} α 1.51, R_{17} β, 2.09; S_{17} α, 1.36; S_{17} β, 2.14); H17 (S_{17}, 3.26; R_{17}, 3.41); H18, 0.73; H19, 1.04; formamide 17-HN (R_{17}, 6.10; S_{17}, 5.56); formamide 17-HCO (R_{17}, 8.03; S_{17}, 8.20); 3-HCO, 8.04.

^{13}C NMR <u>3b</u> (d-chloroform): C1, 36.9; C2, 27.5; C3, 73.8; C4, 42.2; C5, 139.4; C6, 122.6; C7, 31.5; C8, 35.4; C9, 53.9; C10, 37.32; C11, 20.25; C12, 36.2; C13 (R_{17}, 42.8; S_{17}, 42.9); C14 (R_{17}, 52.8; S_{17}, 52.8); C15 (R_{17}, 20.4; S_{17}, 20.5); C16 (R_{17}, 28.8; S_{17}, 28.6); C17 (R_{17}, 62.6; S_{17}, 57.5); C18, 11.7; C19, 19.12; formamide carbonyl (R_{17}, 164.2; S_{17}, 164.3); 3-CHO, 160.4.

^{15}N NMR <u>3b</u> (d-chloroform): formamide S_{17}, 114.2; R_{17}, 120.2.

HRMS (3b): calculated for $C_{21}H_{32}NO_3$ (M+1), 346.24382, observed, 346.2365

Peaks eluted from C18 reverse phase HPLC (acetonitrile/water gradient), 68% ACN (S_{17}) and 70% ACN (R_{17}).

Isonitrile derivatives **2c** and **3c** (identified as compounds **2** and **3** in the Communication) were prepared from compounds **2b** and **3b** as **1d** above. Presence of the isonitrile group was confirmed

in each case by a strong narrow infrared absorbance band at 2138 cm^{-1} in the products after removal of solvent and excess pyridine under vacuum at room temperature.

^{1}H NMR (2c) (d6-DMSO): H1, 1.12, 1.79; H2 1.56, 1.86; H3, 4.77; H4, 1.64, 1.44; H5, 1.27; H6, 1.28, 1.34; H7, 0.96, 1.71; H8, 1.44; H9, 1.09; H11, 1.36, 1.65; H12, 1.77, 2.25; H14, 0.76; H15, 1.36, 1.69; H16, 1.23, 1.80; H17 (S_{17}, 3.82; R_{17}, 3.61); H18, (S_{17}, 0.82; R_{17}, 0.90); H19, 1.10; 3-HCO, 8.31.

^{13}C NMR (2c) (d6-DMSO): C1, 36.5; C2, 27.7; C3, 73.4; C4, 34.3; C5, 44.5; C6, 28.0; C7, 31.7; C8, 36.3; C9, 51.4; C10, 36.0; C11, 23.8; C12, 29.4; C13 (R_{17}, 50.0; $S_{17,}$ 43.6); C14, 53.8; C15 20.7; C16 (R_{17}, 45.1; $S_{17,}$ 35.9); C17 (R_{17}, 62.6; S_{17}, 62.1); C18, 13.0; C19, 12.5; isonitrile C (S_{17}, 156.5; R_{17}, 156.1); 3-CHO, 162.3.

^{15}N NMR (2c) (d6-DMSO): isonitrile S_{17}, 180.0; R_{17}, 193.4.

2c HRMS (identified as compound **2** in the communication**)**: calculated for $C_{21}H_{32}NO_2$ (M+1), 330.2433, observed 330.2417

Peak eluted from C18 reverse phase HPLC (acetonitrile/water gradient), 88% ACN.

^{1}H NMR (3c) (d6-DMSO): H1, 1.21, 1.97; H2 1.70, 1.72; H3, 4.65; H4, 2.42, 2.42; H6, 5.47; H7, 1.72, 2.08; H8, 1.53; H9, 1.10; H11, 1.55, 1.72; H12, 1.28, 1.84; H14, 1.37; H15, (S_{17}, 2.07, 1.72; R_{17}, 2.36, 1.84); H16, (S_{17}, 1.28, 1.84; R_{17}, 1.22, 1.78); H17 (R_{17}, 3.60; S_{17}, 3.82); H18, 0.86; H19, 0.90; 3-HCO, 8.27.

^{13}C NMR (3c) (d6-DMSO): C1, 36.9; C2, 23.7; C3, 73.6; C4, 37.7; C5, 140.4; C6, 122.8; C7, 31.9; C8, 32.1; C9, 50.1; C10, 33.7; C11, 20.5; C12, 35.7; C13 (S_{17}, 45.5; $R_{17,}$ 43.5); C14, 50.2; C15 (S_{17}, 31.8; $R_{17,}$ 31.4); C16 (S_{17}, 35.8; $R_{17,}$ 36.8); C17 (S_{17}, 62.7; R_{17}, 62.3); C18, (S_{17}, 16.7; $R_{17,}$ 12.6); C19, 19.5; isonitrile C (S_{17}, 156.2; R_{17}, 156.9); 3-CHO, 162.3.

^{15}N NMR (3c) (d6-DMSO): isonitrile S_{17}, 183.9; R_{17}, 179.4.

3c HRMS calculated for $C_{20}H_{30}NO$ (M+1, free 3-OH)**,** 300.2327, found 300.2308

Peak eluted from C18 reverse phase HPLC (acetonitrile/water gradient), 80% ACN.

3β-formyl-(R,S)-(7,17-bis-amidoformyl)androst-5,6-ene **4a** was prepared as with compounds **1-3**, starting from androstene-3β-hydroxy-5,6-dehydro-7,17-dione (CAS 566-19-8, Steraloids, Inc., Newport, RI).

^{1}H NMR 4a (d_6-acetone): H1, 1.26, 1.94; H2 1.68, 1.88; H3, 4.87; H4, 1.95, 1.92; H6, 5.80; H7, 4.49; H8, 1.87; H9, 1.35; H11, 1.51, 1.79; H12, 1.34, 1.83; H14, 1.47; H15, 1.61, 2.11; H16 α 1.31, 1.29; β, 1.81; H17 (epimer 1, 3.45; epimer 2, 4.01); H18, 0.87; H19, 1.1; formamide 7-NH, 7.37; 17-NH, 7.17; formamide 7-HCO 8.18; 17-HCO, 8.24; 3-HCO, 8.19.

^{13}C NMR 4a (d_6-acetone): C1, 33.1; C2, 24.1; C3, 73.3; C4, 33.2; C5, 142.6; C6, 127.2; C7, 49.3; C8, 37.7; C9, 48.6; C10, 34.6; C11, 20.6; C12, 36.8; C13 43.7; C14, 52.8; C15 28.2; C16 (epimer 1, 37.1; epimer 2,, 36.1); C17 (epimer 1, 56.9; epimer 2, 62.9); C18, 11.5; C19, 17.8; formamide carbonyl (7-, 160.3; 17-, 160.8); 3-CHO, 162.3.

^{15}N NMR 4a (d_6-acetone): 7-N, 135.3; 17-N, 125.6

3β-formyl-(R,S)-(7,17-bis-isonitrilo)androst-5,6-ene **4b** The crude 7,17-diformamide **4a** was dried over P_2O_5 in vacuum and used to prepare the isonitrile as above. After 3x extraction of the crude neutralized reaction mixture with diethyl ether, the dehydration of **4a** to **4b** was found to be essentially complete by NMR. Based on integration of the C7 1H peak, the major isonitrile at that position is produced in ~4:1 over the minor epimer. NOEs between 7H, 9H and 14H that the major epimer isonitrile at the 7 position is in the *R*-configuration. At the 17 position, the isonitrile is ~10:1 *S*-configuration, based on NOEs between the 17, 15 and 16 (but not 18-CH_3) protons. Some evidence of dehydration by loss of the 3-hydroxyl group is present in the isonitrile spectrum, in the form of new vinyl protons (presumably at 3- and 4- positions). The presence of the isonitrile was confirmed by IR spectroscopy, with a strong absorption band at 2138 cm^{-1}.

^{1}H NMR **4b** (d_6-DMSO): H1, 1.14, 1.87; H2 1.79, 1.97; H3, 4.77; H4, 1.87, 1.87; H6, 5.37 (R), 5.28 (S); H7, 4.30 (R), 4.28 (S); H8, 1.93; H9, 1.16; H11, 1.47, 1.75; H12, 1.27, 1.84; H14, 1.42; H15, 1.83, 2.30; H16, 1.28, 1.84 (S), 1.27, 1.82 (R); β, 1.81; H17, 3.65 (S), 3.74 (R); H18, 0.95 (S), 0.91 (R); H19, 1.06; 3-HCO, 8.27.

^{13}C NMR **4b** (d_6-DMSO): C1, 33.0; C2, 25.3; C3, 73.4; C4, 33.0; C5, 144.4; C6, 119.7 (R), 117.8 (S); C7, 55.3 (R), 55.7 (S); C8, 39.3; C9, 46.7; C10, 34.6; C11, 20.5; C12, 35.3; C13, 44.2; C14, 52.8; C15 29.4; C16, 35.4 (S), 35.3 (R); C17, 61.9 (S), 61.9 (R)); C18, 12.7 (S), 12.6 (R); C19, 18.5; 3-CHO, 162.8, 7-N**C**, 156.6, 17-N**C**, 156.6.

^{15}N NMR **4b** (d_6-DMSO): 7-**N**C, 185.0; 17-**N**C, 177.4

HRMS for $C_{22}H_{28}N_2O_2$ (M+1), 353.2230, found 353.2224.

Peak eluted from C18 reverse phase HPLC (acetonitrile (ACN)/water gradient), 70% ACN.

***) Note on NMR assignments and spectra:** All NMR assignments are numbered according to the accepted IUPAC system for gonane and steranes, shown in the structure above for compound **4**. Relevant NMR spectra used for resonance assignment of all isonitrile compounds 1-4 are deposited as Supplementary Data 1.

NMR spectroscopy. All two-dimensional NMR experiments used for resonance and stereochemical assignments were performed on a Bruker NEO spectrometer at the Landsman Research Facility (Brandeis Univ.) operating at 800.13 MHz (^{1}H), 201.19 MHz (^{13}C) and 81.08 MHz (^{15}N). All ^{1}H and ^{13}C chemical shifts are reported in ppm relative to tetramethylsilane; ^{15}N shifts are reported in ppm relative to anhydrous ammonia. For assigning ^{1}H and ^{13}C correlations, ^{1}H,^{13}C-HSQC and HMBC experiments were performed. Formamide ^{15}N resonances were assigned using natural abundance ^{1}H,^{15}N-HSQC, and ^{1}H,^{15}N-HMBC used for assigning isonitrile ^{15}N resonances. Stereochemistry at reaction centers was established by analysis of ^{1}H,^{1}H NOESY experiments, based on the known stereochemistry of steroid starting materials, except in the case of **1c**, in which the crystal structure of the CYP17A1-**1c** complex provided the stereochemical relationship at C_{20}.

Infrared spectroscopy. Infrared spectra were recorded on a diamond anvil-equipped Nicolet FTIR spectrometer.

High-resolution mass spectrometry. High-resolution mass spectra were obtained at the Brandeis University interdepartmental mass spectrometry facility on a Bruker timsTOF Pro mass spectrometer operating in positive ion mode in tandem with liquid chromatographic separation of analytes.

S2) Ligand binding and inhibition assays

Reduction and reoxidation assays. Reduction of the isonitrile-bound P450 complex was observed by taking a baseline absorbance reading from 400 to 500 nm using a UV-visible scanning spectrophotometer, of a 1cm quartz cuvette containing 1 μM P450, with the selected isonitrile compound at a saturating concentration (*i.e.,* ≥ concentration needed to reach ΔA_{max}), in the same buffer used for binding assays (100 mM potassium phosphate buffer (pH 7.4), 20% glycerol, and 100 mM sodium chloride). A small quantity of sodium dithionite (spatula tip-full) was then added to the cuvette, and the spectra were recorded periodically over time to observe the formation of peaks indicating the reduction (~426 and 456 nm) and subsequent reoxidation (~435 nm) of the complex. The total time course for Figure 6B was 103 minutes and 37 seconds.

CYP17A1 Inhibition assay. (Adapted from DeVore, NM, Scott, EE. (2012). *Nature, 482*(7383), 116-119.) Metabolic activity of CYP17A1 was evaluated by measuring 17α-hydroxylation of progesterone as detected by HPLC with UV detection at 240 nm. A 1:4 ratio of CYP17A1 to recombinant NADPH-cytochrome P450 reductase was mixed and incubated on ice for 20 minutes. This mixture was added to buffer (50 mM Tris, pH 7.4, 5 mM $MgCl_2$) containing 11.5 mM progesterone and either abiraterone (0-1 μM) or **1c** (0-50 μM). Reaction vials were warmed to 37 °C for three minutes, then catalysis was initiated by adding NADPH to a final concentration of 1 mM. After 10 minutes, metabolism was quenched by adding 300 μL of 20% trichloroacetic acid and placed on ice. The reaction vials were centrifuged to pellet the precipitated protein, then the supernatant was injected onto a C18 column (Phenomenex, Luna, 50 x 4.6 mm) for HPLC. The 30-minute HPLC method ran at 0.8 mL/min and started with a mobile phase of 60% acetonitrile, 40% water with 0.2% acetic acid for five minutes, increased to 80% acetonitrile over 10 minutes, held at 80% acetonitrile for five minutes, 100% acetonitrile for five minutes, then returned to 60% acetonitrile to prepare for the next sample. Metabolite elution was normalized to β-estradiol as an internal standard. A standard curve of known product concentrations was used to convert normalized area under curve to amount of product produced. A four-parameter variable-slope equation ($Y=Y_{min} + (Y_{max}-Y_{min})/(1+(IC_{50}/X)$^(Hill Slope))) was used to fit the data and calculate IC_{50} values for inhibitors using GraphPad Prism 9.

CYP3A4 nifedipine metabolism assay. (Adapted from Bart, AG, Scott, EE. (2017). *J. Biol. Chem.,* 292(51), 20818–20833.) Metabolic activity of CYP3A4 was evaluated by measuring metabolism of nifedipine to dehydronifedipine, as detected by HPLC with UV detection at 254 nm. Reactions were carried out in amber microcentrifuge tubes (NFP is light sensitive) in a final volume of 150 µl. A 1:2 ratio of CYP3A4 to recombinant NADPH-cytochrome P450 reductase was mixed and incubated at room temperature for 20 minutes. This mixture was added to buffer (40 mM HEPES, 30 mM $MgCl_2$, pH 7.4) containing 0.1 mM NFP and increasing concentrations of **1c** (0-100 μM). Reaction vials were warmed to 37 °C for three minutes, then catalysis was initiated by adding NADPH to a final concentration of 1 mM. After 20 minutes, metabolism was quenched by adding 50 µL acetonitrile and placed on ice. The reaction vials were centrifuged at 5000 xg for 5 minutes to pellet the precipitated protein, then the supernatant was injected onto a C18 column (Phenomenex, Luna, 50 x 4.6 mm) for HPLC. Separation on HPLC was obtained using a mobile phase of 45%/55% water/methanol for 40 minutes. A standard curve of known product concentrations was used to convert the area under the curve to the amount of product produced.

S3) Crystallization of 1-CYP17A1 complex and X-ray diffraction

Protein Crystallization and Structure Determination. CYP17A1 was saturated with 3β-formyl-5α pregnanolone-20(*R*)-isonitrile (verified by 430 nm Soret peak) and prepared for crystallization via hanging

drop vapor diffusion. A protein solution of 30 mg/mL CYP17A1 in 50 mM Tris-HCl (pH 7.4), 20% glycerol, 500 mM NaCl, and 0.2% Emulgen 913 was equilibrated against 0.1 M Tris-HCl (pH 8.5), 0.25 M $LiSO_4$, 30% PEG 3350, and 7% sucrose at 22 degrees C. Crystals appeared after 48 hours. Crystals were cryoprotected in mother liquor supplemented with 24% glycerol and flash cooled in liquid nitrogen. Diffraction data was collected at 100 K at the Standford Synchrotron Radiation Laboratory beamline 12-2. Data were processed to 2.2 Angstroms using XDS[1] and Scala[2]. The structure was solved by molecular replacement using PHASER[3] with a search model based on CYP17A1 complexed with 3-keto-5a-abiraterone (PDB 6WW0). Iterative model building and structure refinement were accomplished using COOT[4] and Phenix.refine[5]. Validation of this structure was performed in Phenix[5]. X-ray data statistics are provided in Table S1. All figures were made using PyMOL[6]. Full coordinates are available as Supplemental Data 2.

Table S1. X-ray data collection, refinement, and validation statistics. Highest-resolution shell shown in parentheses. Software used for structure refinement and visualization are referenced below.

	**CYP17A1 bound to 3β-formyl-5α-pregnenolone-(R) C20-isonitrile**
**Data collection**	
Space group	$P2_12_12_1$
Cell dimensions	
A, *b*, *c* (Å)	86.13, 152.27, 171.59
[illegible] (°)	90.00, 90.00, 90.00
Resolution (Å)*	2.20
Redundancy*	13.4 (12.5)
R_{pim}*	0.075 (1.453)
Mn(I/sd)*	6.8 (0.9)
CC ½*	0.997 (0.382)
Completeness* (%)	99.6 (99.1)
Total Reflections*	1,536,253 (206,362)
Unique Reflections*	114,766 (16,489)
**Refinement**	
Resolution (Å)	38.96 - 2.20
No. reflections	113,087
R_{work} / R_{free}	0.216 / 0.259
Number of non-hydrogen atoms / B factor	
Protein	14871 / 55.8
Ligand	156 / 62.3
Heme	172 / 45.3
Solvent	268 / 49.6
R.M.S deviations	
Bond lengths (Å)	0.005
Bond angles (º)	0.73
Coordinate error (Maximum-likelihood) (Å)	0.30
Ramachandran plot: preferred/allowed/outliers (%)	96.7 / 3.2 / 0.11

S4) Supplementary References

1. Kabsch, W., XDS. *Acta Crystallographica Section D: Biological Crystallography* **2010,** *66* (2), 125-132.
2. Evans, P., Scaling and assessment of data quality. *Acta Crystallographica Section D* **2006,** *62* (1), 72-82.
3. McCoy, A. J.; Grosse-Kunstleve, R. W.; Adams, P. D.; Winn, M. D.; Storoni, L. C.; Read, R. J., Phaser crystallographic software. *Journal of Applied Crystallography* **2007,** *40* (4), 658-674.
4. Emsley, P.; Lohkamp, B.; Scott, W. G.; Cowtan, K., Features and development of Coot. *Acta Crystallographica Section D* **2010,** *66* (4), 486-501.
5. Adams, P. D.; Afonine, P. V.; Bunkoczi, G.; Chen, V. B.; Davis, I. W.; Echols, N.; Headd, J. J.; Hung, L.-W.; Kapral, G. J.; Grosse-Kunstleve, R. W.; McCoy, A. J.; Moriarty, N. W.; Oeffner, R.; Read, R. J.; Richardson, D. C.; Richardson, J. S.; Terwilliger, T. C.; Zwart, P. H., PHENIX: a comprehensive Python-based system for macromolecular structure solution. *Acta Crystallographica Section D* **2010,** *66* (2), 213-221.
6. DeLano, W. L. *The PyMOL Molecular Graphics System*, version 1.8.6.2; Schrodeinger, LLC: New York, 2017.
