# Supplementary material for: Selective steroidogenic cytochrome P450 haem iron ligation by steroid-derived isonitriles
Source: Commun Chem. 2023 Sep 2;6:183. doi: 10.1038/s42004-023-00994-3 (PMC10475101; doi:10.1038/s42004-023-00994-3)
Supplement: Supplementary file 2 — Description of Additional Supplementary Files [file 42004_2023_994_MOESM2_ESM.pdf]

Description of Additional Supplementary Files

File name: Supplementary Data 1

Description: NMR spectra used for assignment of compounds

File name: Supplementary Data 2

Description: PDB format coordinates of described complex

File name: Supplementary Data 3

Description: Excel format numerical data used to derive all graphs in figures.
