# Supplementary material for: Selective steroidogenic cytochrome P450 haem iron ligation by steroid-derived isonitriles
Source: Commun Chem. 2023 Sep 2;6:183. doi: 10.1038/s42004-023-00994-3 (PMC10475101; doi:10.1038/s42004-023-00994-3)
Supplement: Supplementary file 3 — Supplementary Data 1 [file 42004_2023_994_MOESM3_ESM.pdf]

newred 1 1 /Users/pochapsk/brukerdata/steroid_data

t_1h C6D6 /opt/nmrdata/user/ssp/ ssp_auto 8

pregnalone-derived isonitrile **1c** (both C20 epimers present)

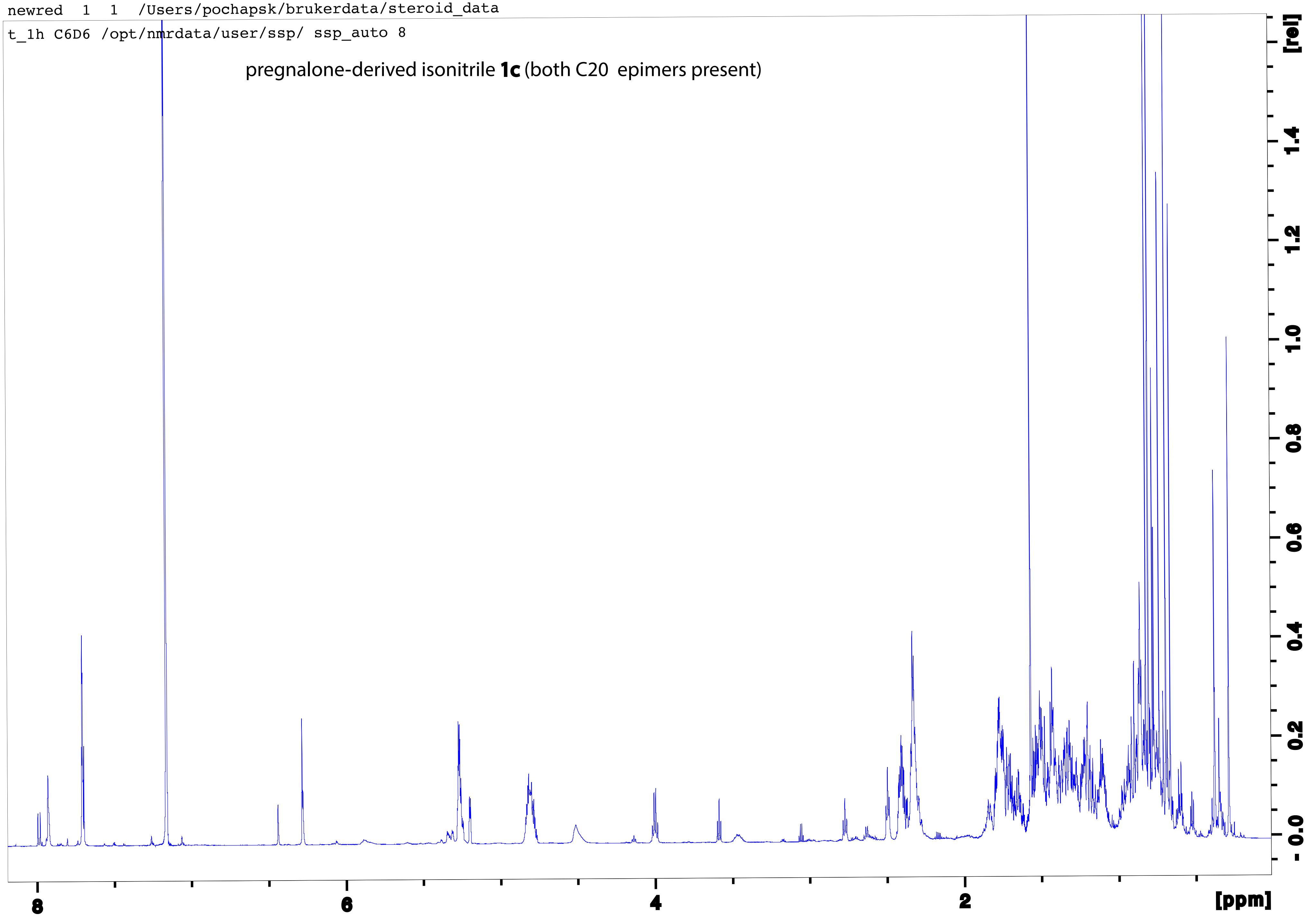

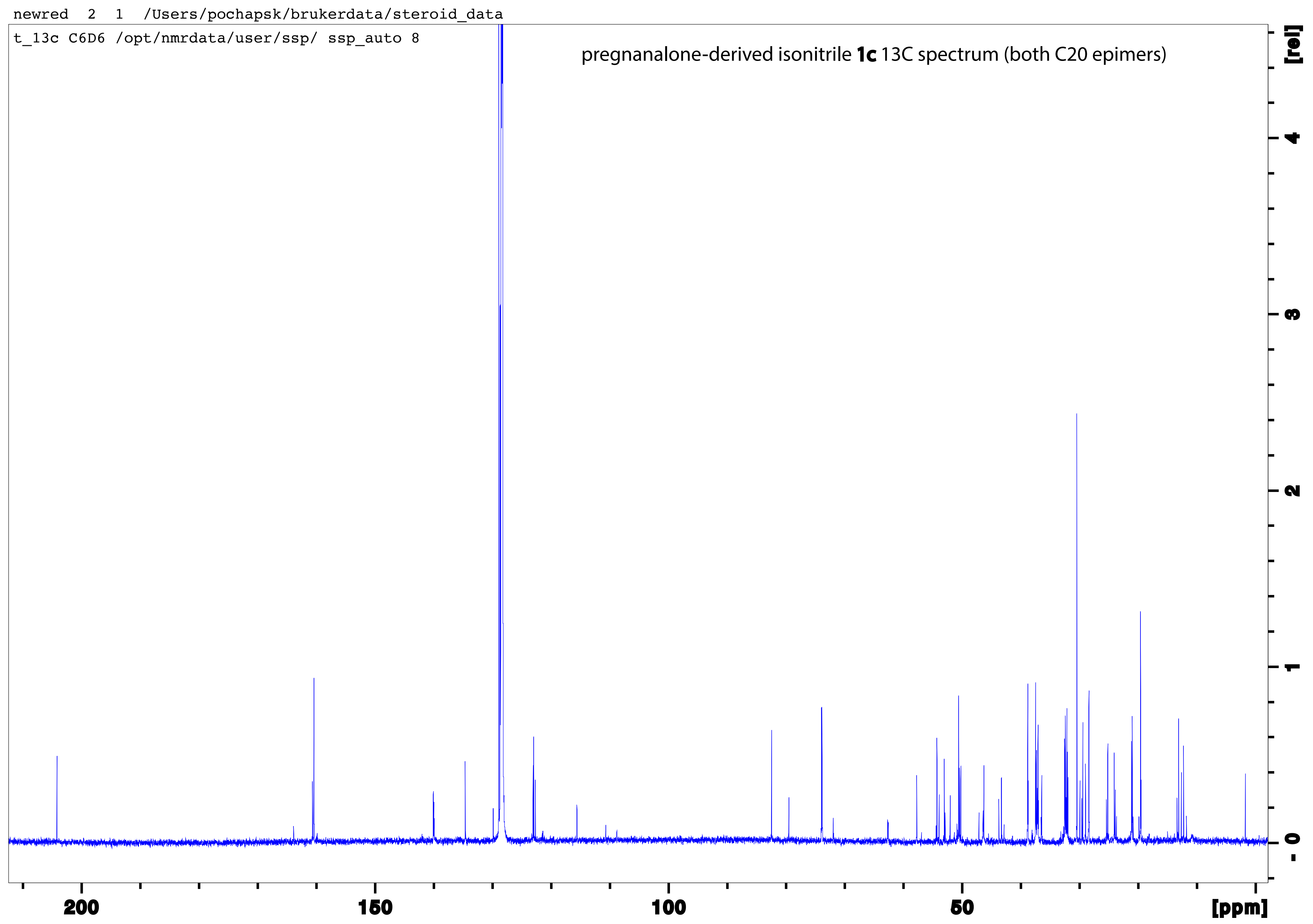
newred 2 1 /Users/pochapsk/brukerdata/steroid_data
t_13c C6D6 /opt/nmrdata/user/ssp/ ssp_auto 8
pregnanalone-derived isonitrile **1c** 13C spectrum (both C20 epimers)
200
150
100
50
[ppm]
- 0
1
2
3
4
[rel]

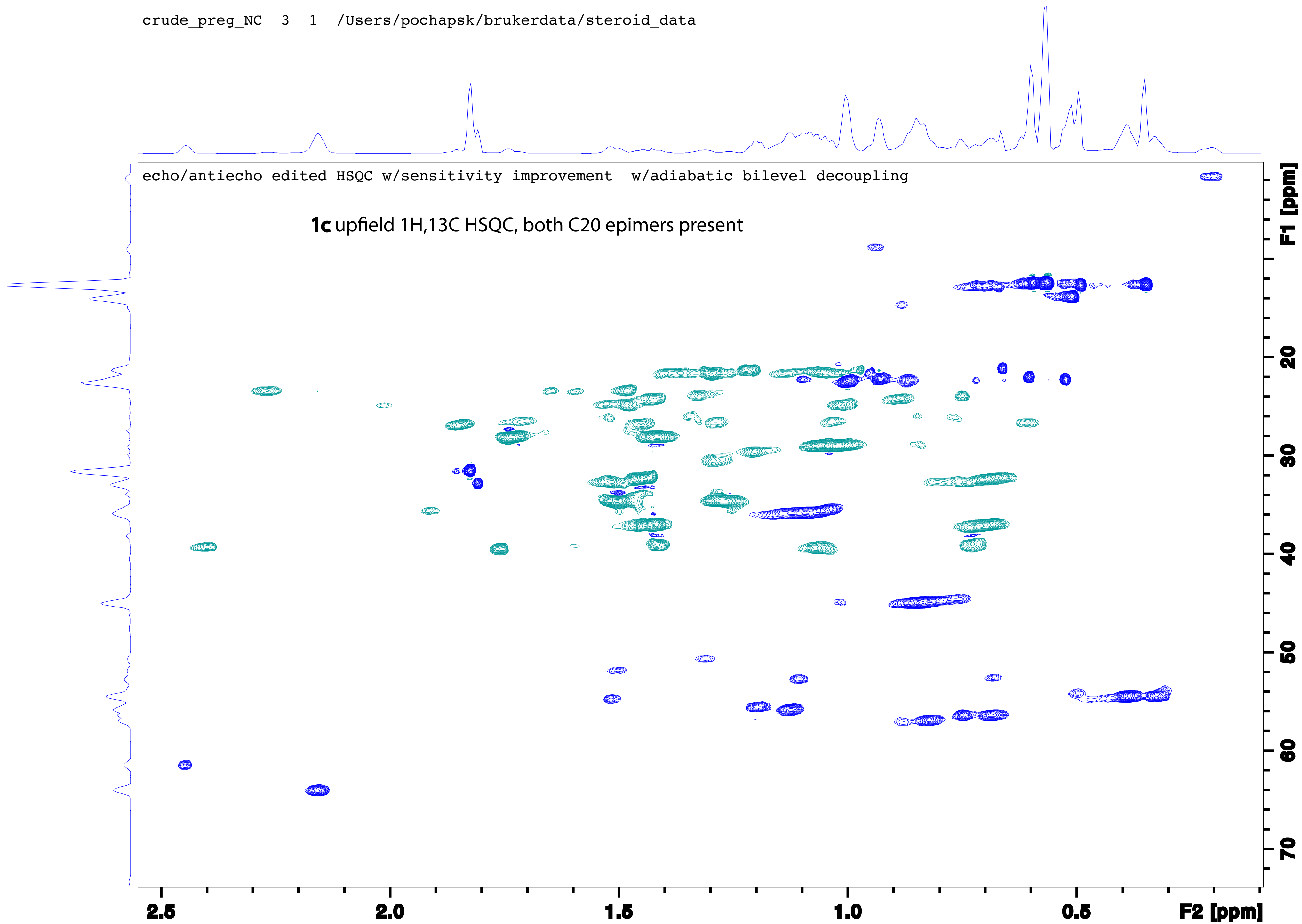
crude_preg_NC 3 1 /Users/pochapsk/brukerdata/steroid_data
echo/antiecho edited HSQC w/sensitivity improvement w/adiabatic bilevel decoupling
1c upfield 1H,13C HSQC, both C20 epimers present
F1 [ppm]
20
30
40
50
60
70
2.5
2.0
1.5
1.0
0.5
F2 [ppm]

crude_preg_NC 3 1 /Users/pochapsk/brukerdata/steroid_data

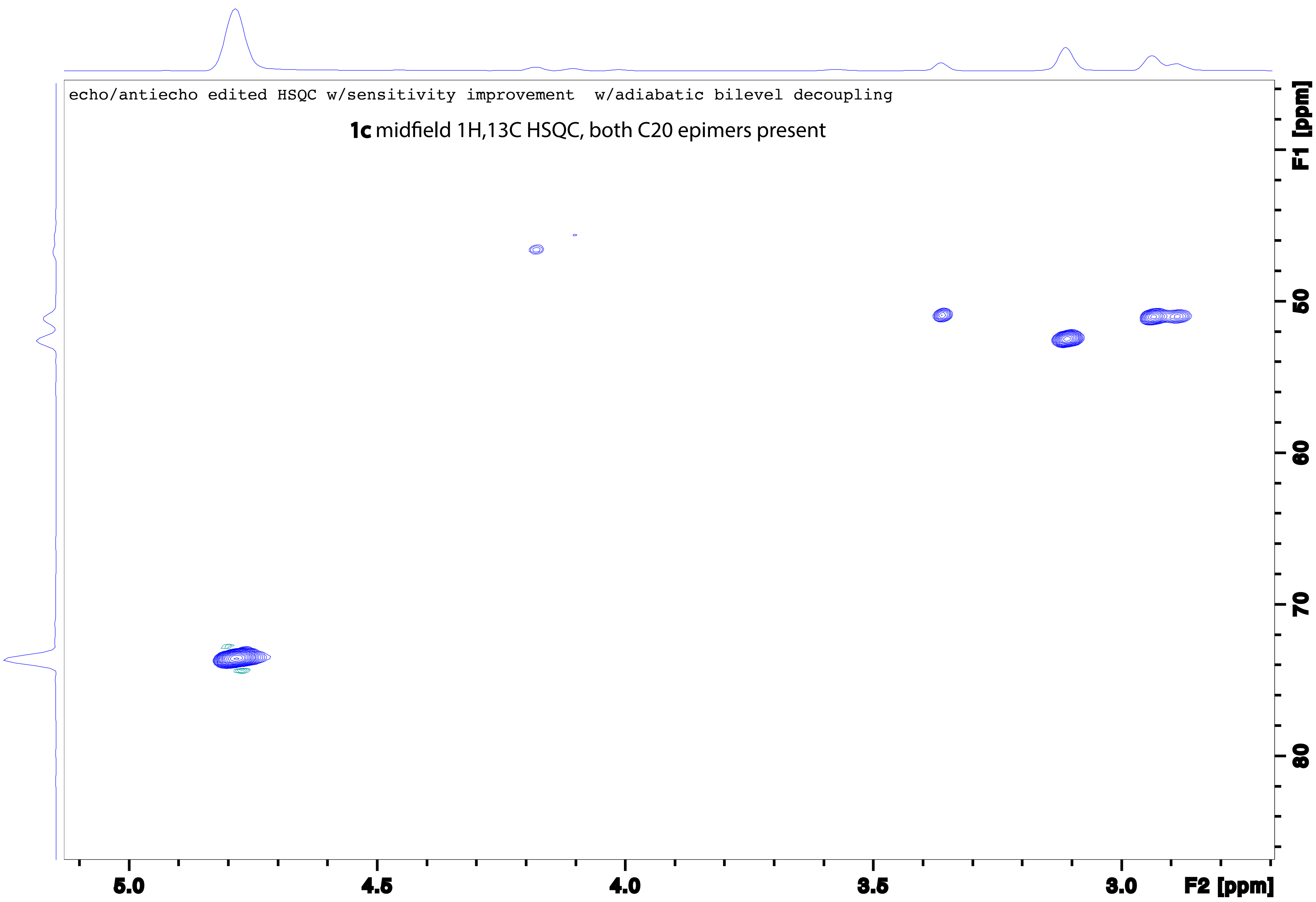

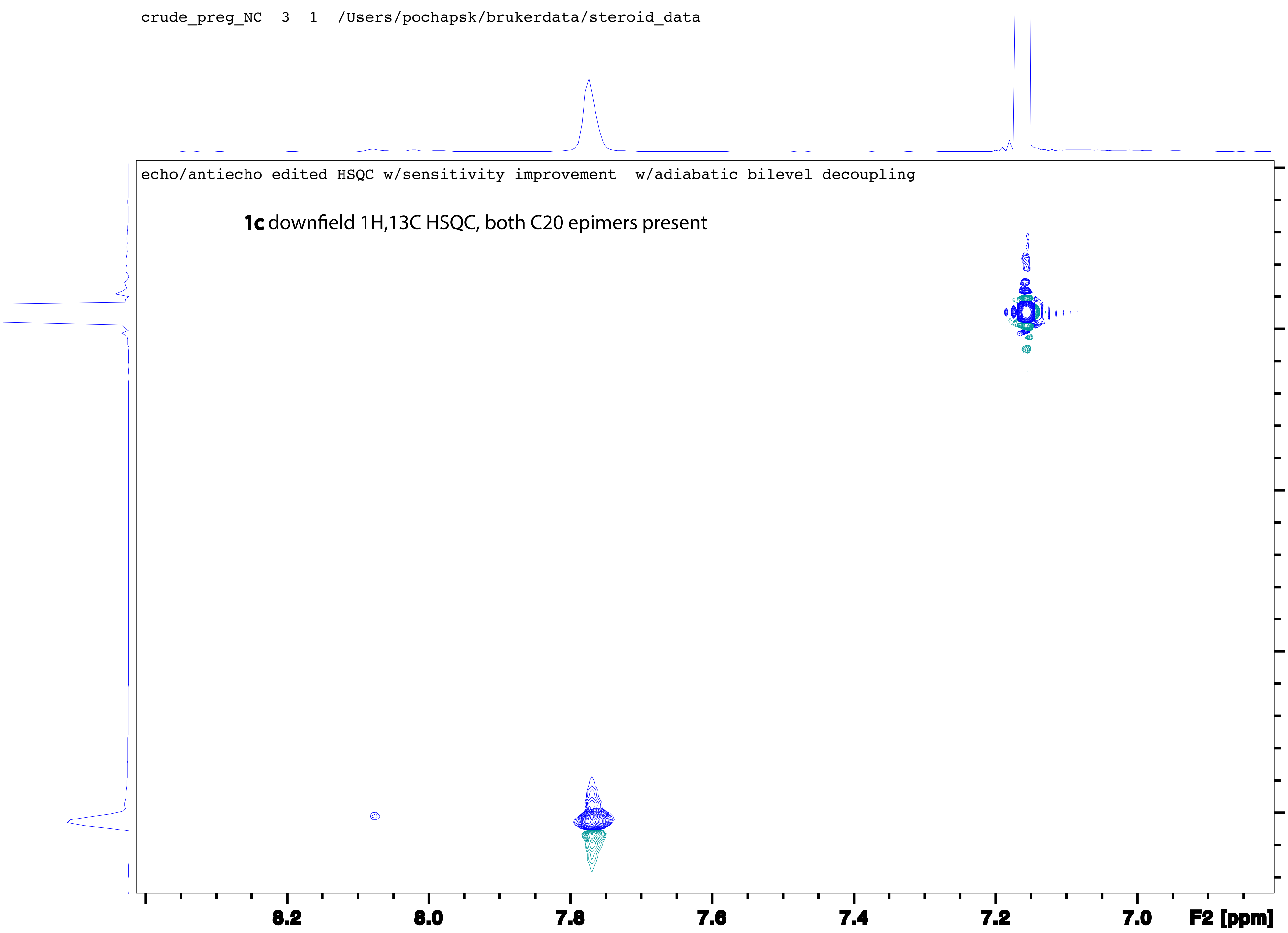

crude_preg_NC 3 1 /Users/pochapsk/brukerdata/steroid_data
echo/antiecho edited HSQC w/sensitivity improvement w/adiabatic bilevel decoupling
1c downfield 1H,13C HSQC, both C20 epimers present
F1 [ppm]
130
140
150
160
8.2
8.0
7.8
7.6
7.4
7.2
7.0
F2 [ppm]

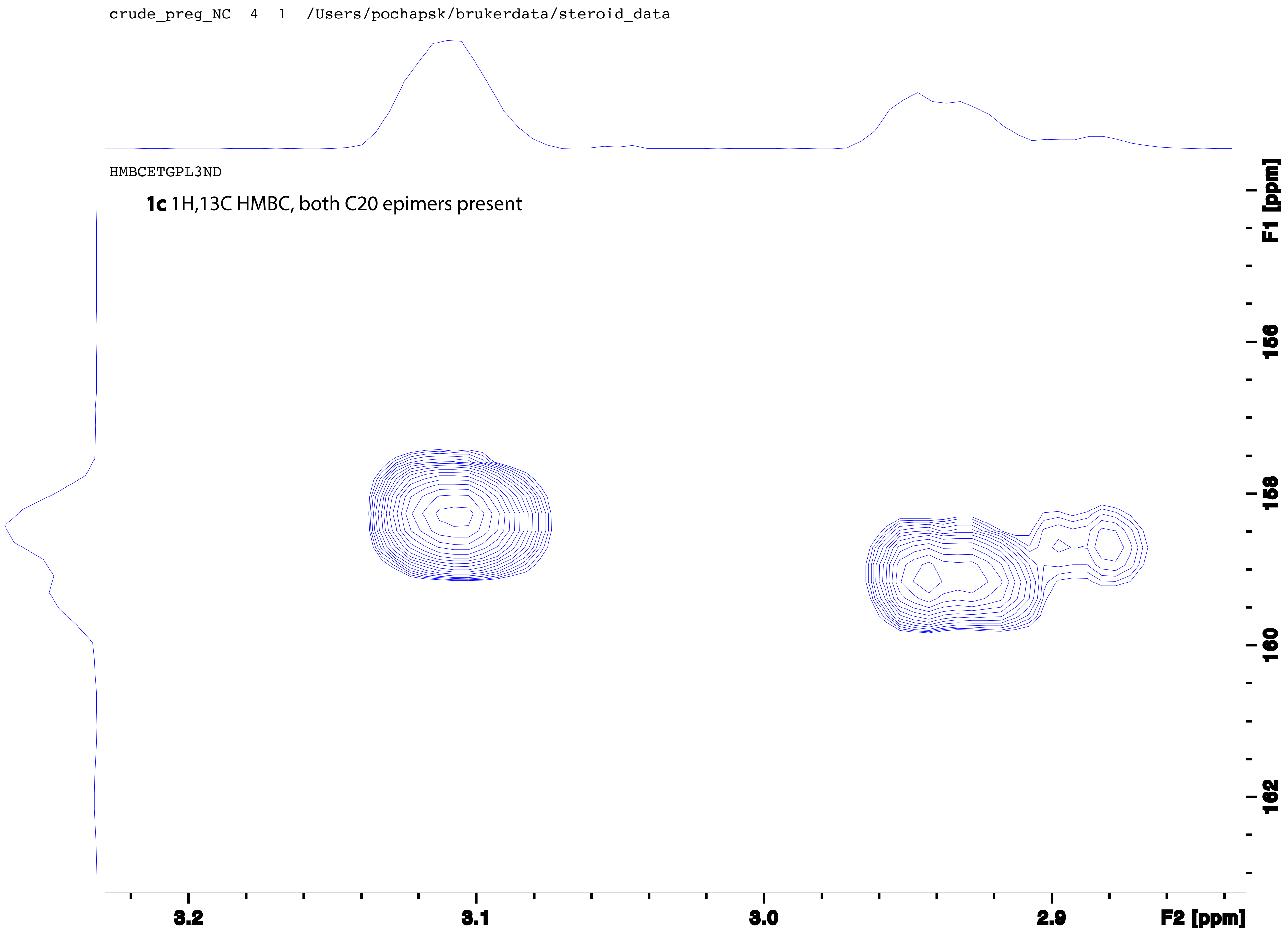
crude_preg_NC 4 1 /Users/pochapsk/brukerdata/steroid_data
HMBCETGPL3ND
1c 1H,13C HMBC, both C20 epimers present
F1 [ppm]
156
158
160
162
3.2
3.1
3.0
2.9
F2 [ppm]

juneyellow 1 1 /Users/pochapsk/brukerdata

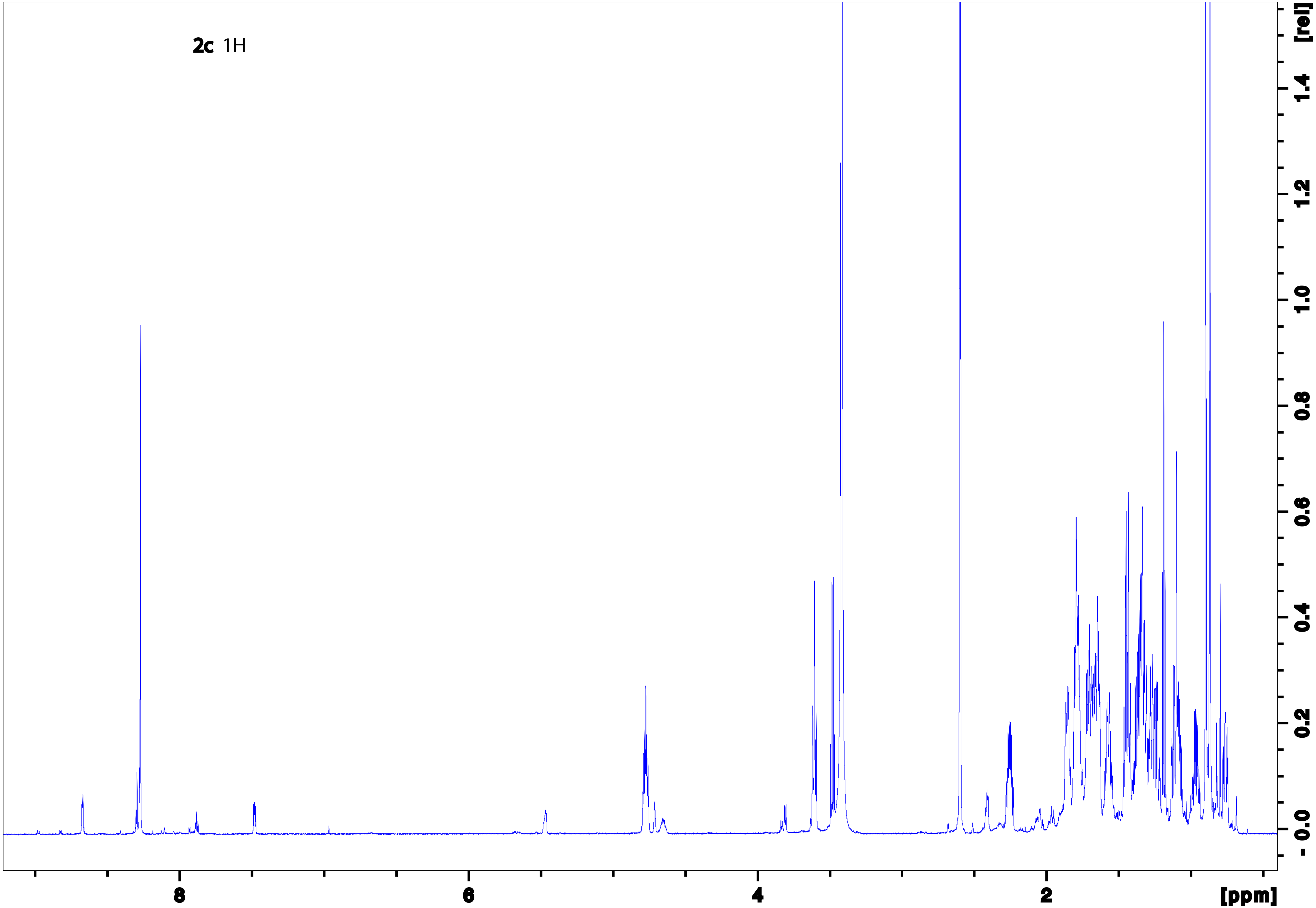

juneyellow 2 1 /Users/pochapsk/brukerdata

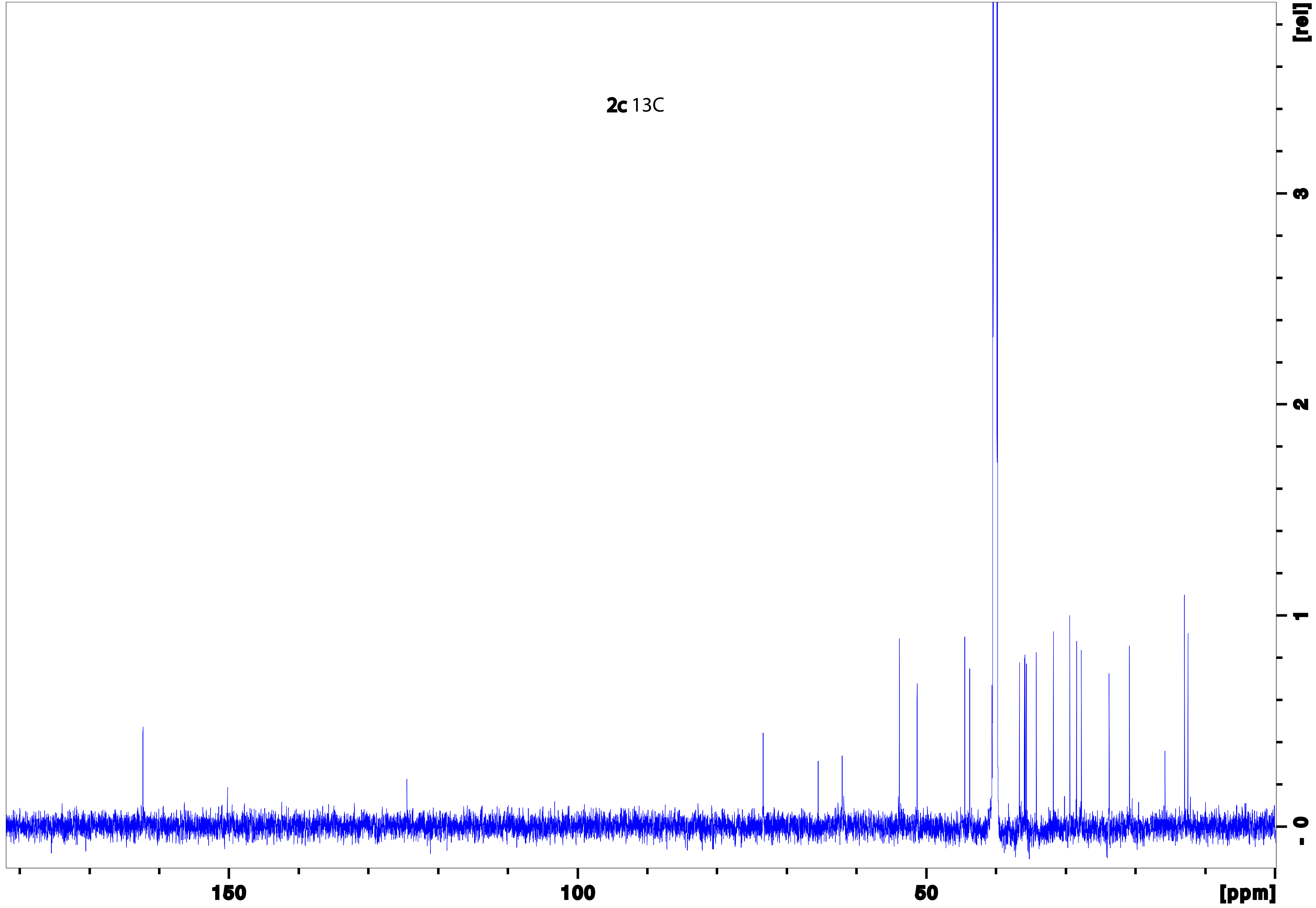

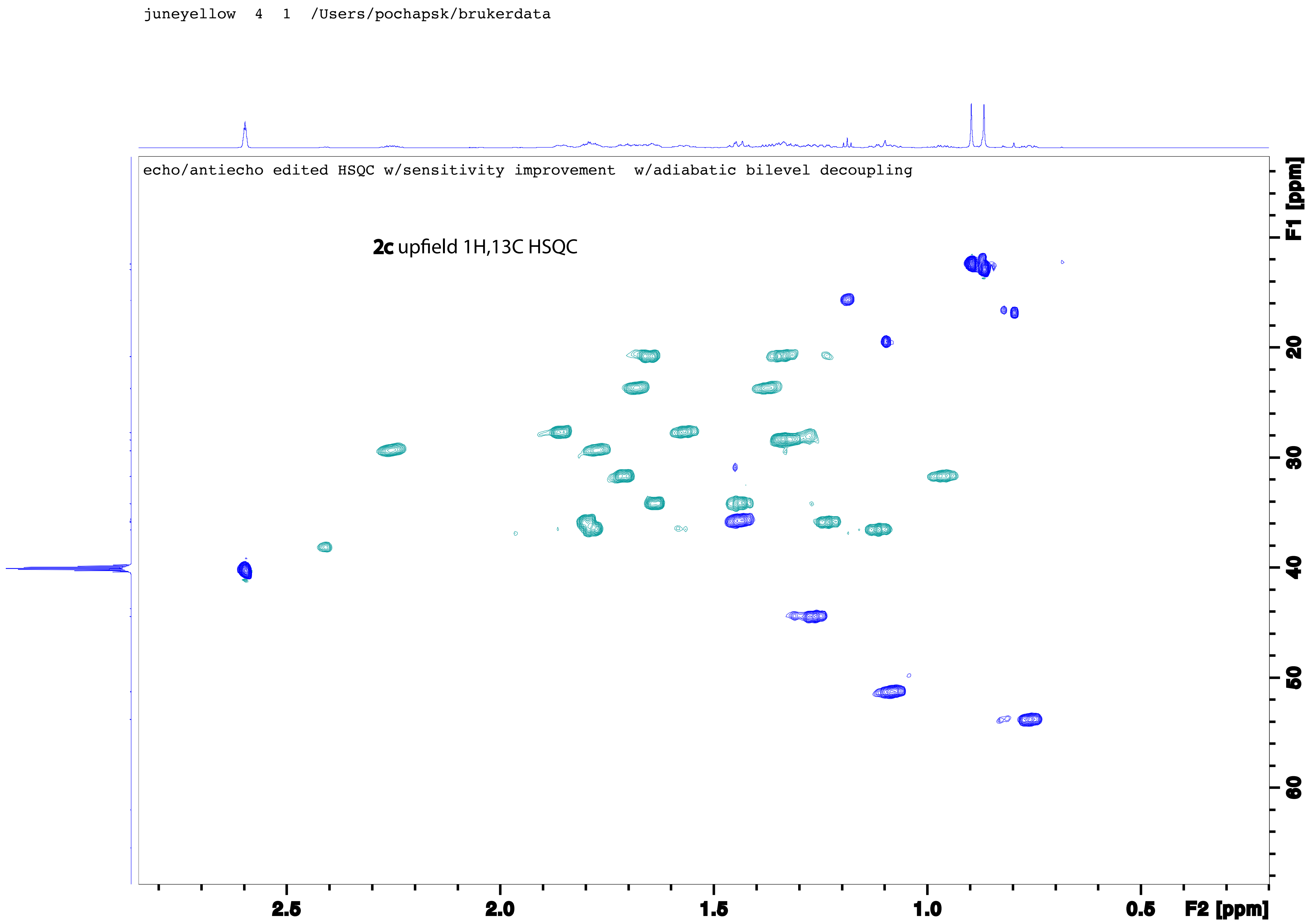
juneyellow 4 1 /Users/pochapsk/brukerdata
echo/antiecho edited HSQC w/sensitivity improvement w/adiabatic bilevel decoupling
2c upfield 1H,13C HSQC
F1 [ppm]
20
30
40
50
60
2.5
2.0
1.5
1.0
0.5
F2 [ppm]

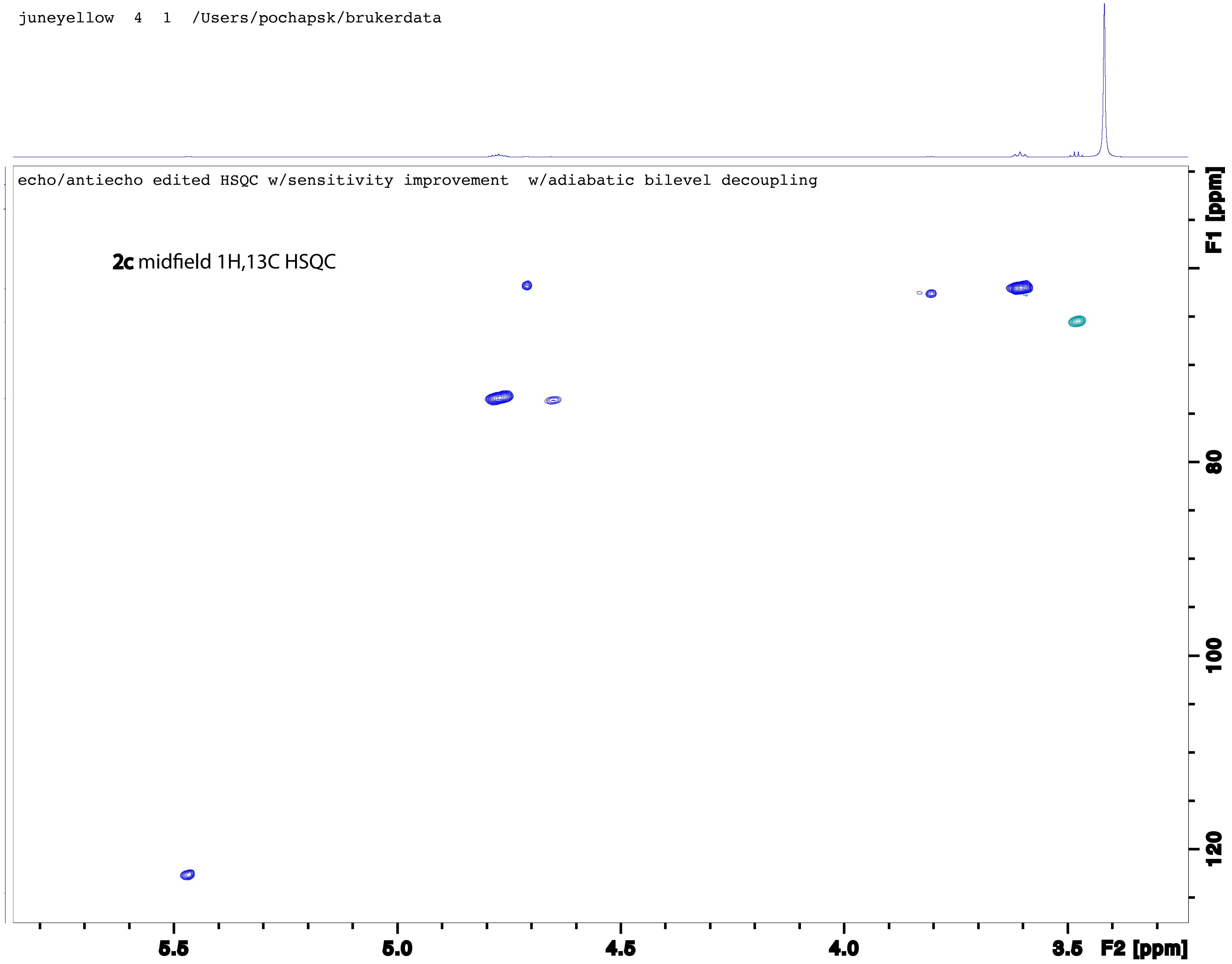
juneyellow 4 1 /Users/pochapsk/brukerdata
echo/antiecho edited HSQC w/sensitivity improvement w/adiabatic bilevel decoupling
2c midfield 1H,13C HSQC
F1 [ppm]
80
100
120
5.5
5.0
4.5
4.0
3.5
F2 [ppm]

juneyellow 4 1 /Users/pochapsk/brukerdata

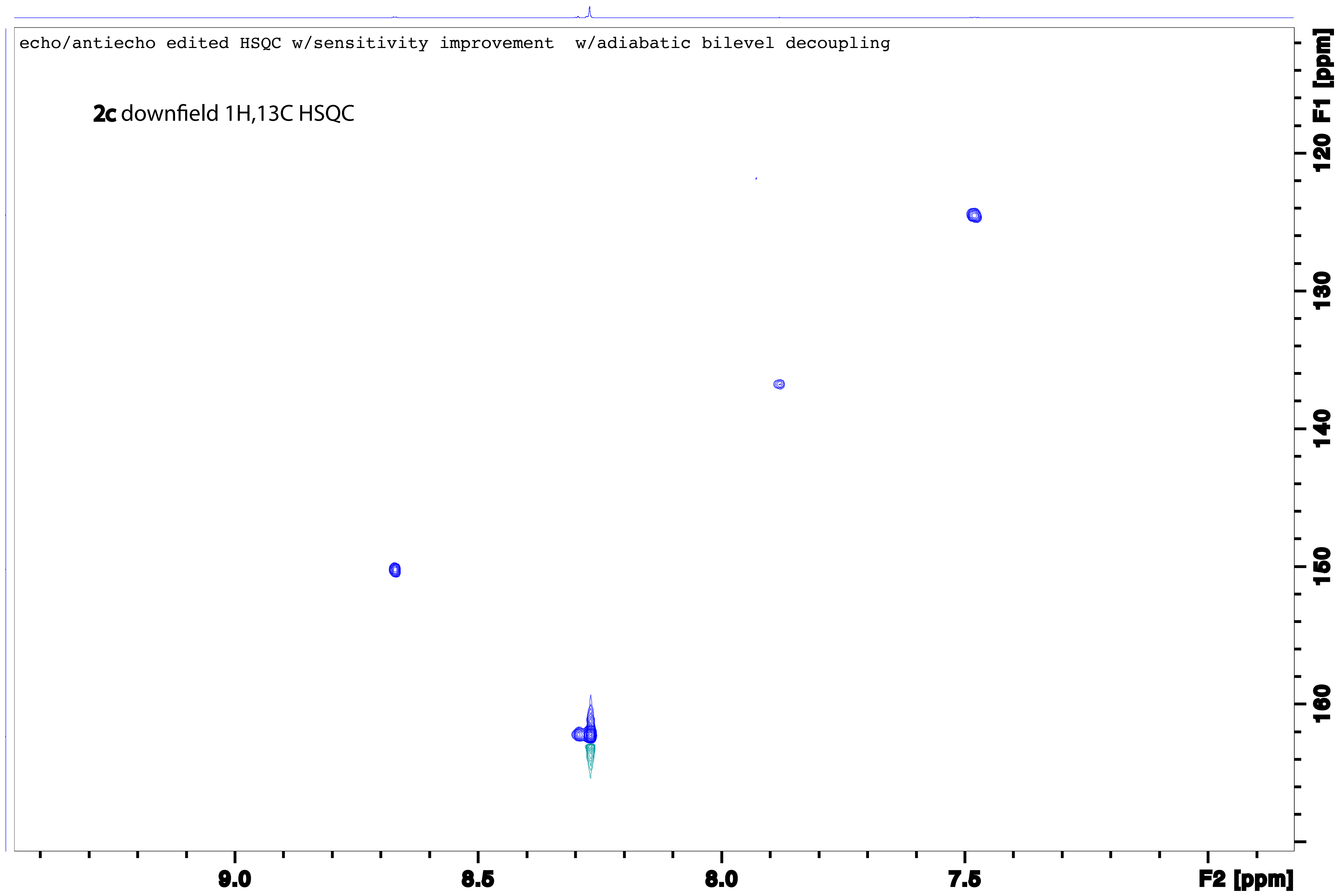

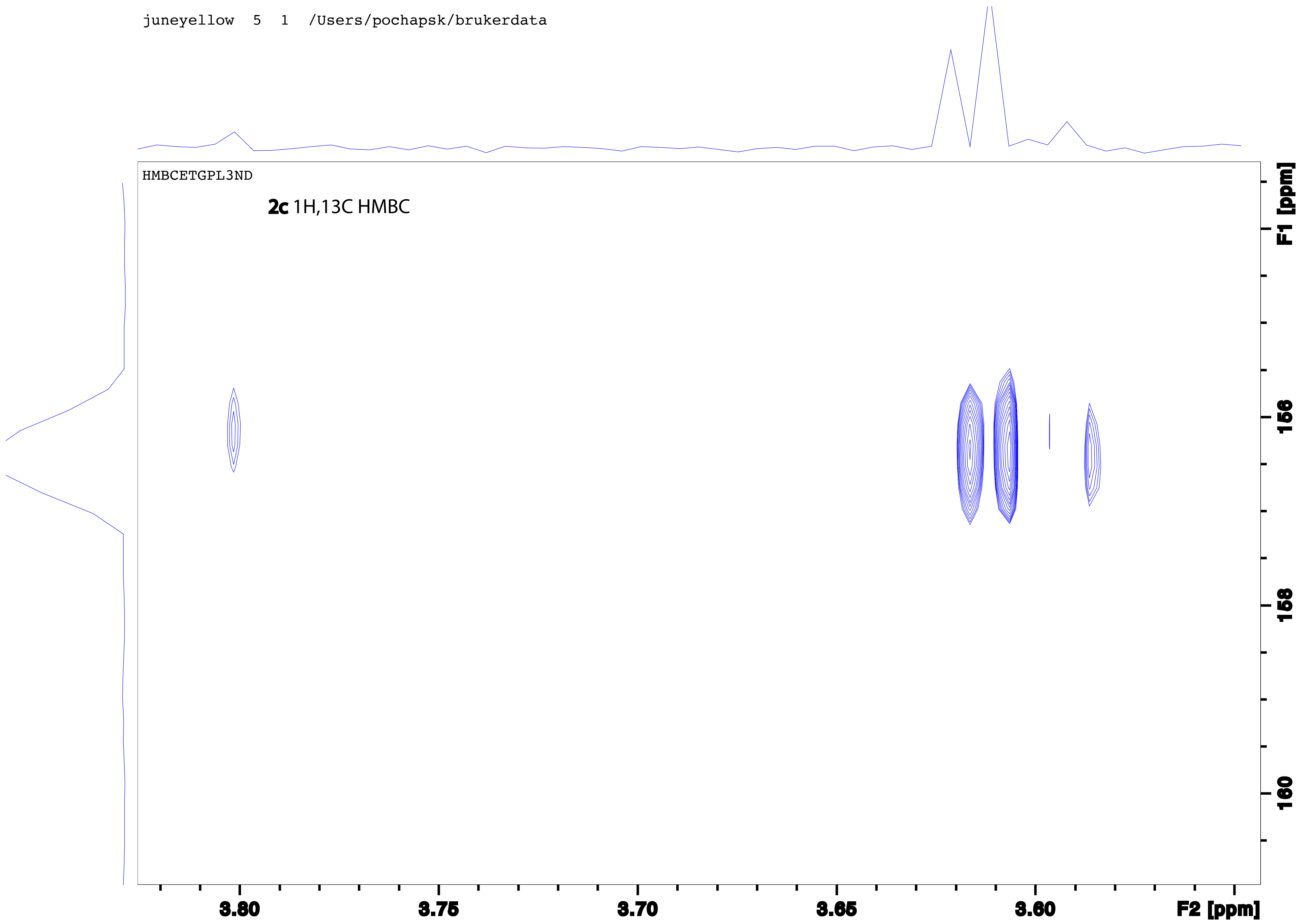
juneyellow 5 1 /Users/pochapsk/brukerdata
HMBCETGPL3ND
2c 1H,13C HMBC
F1 [ppm]
156
158
160
3.80
3.75
3.70
3.65
3.60
F2 [ppm]

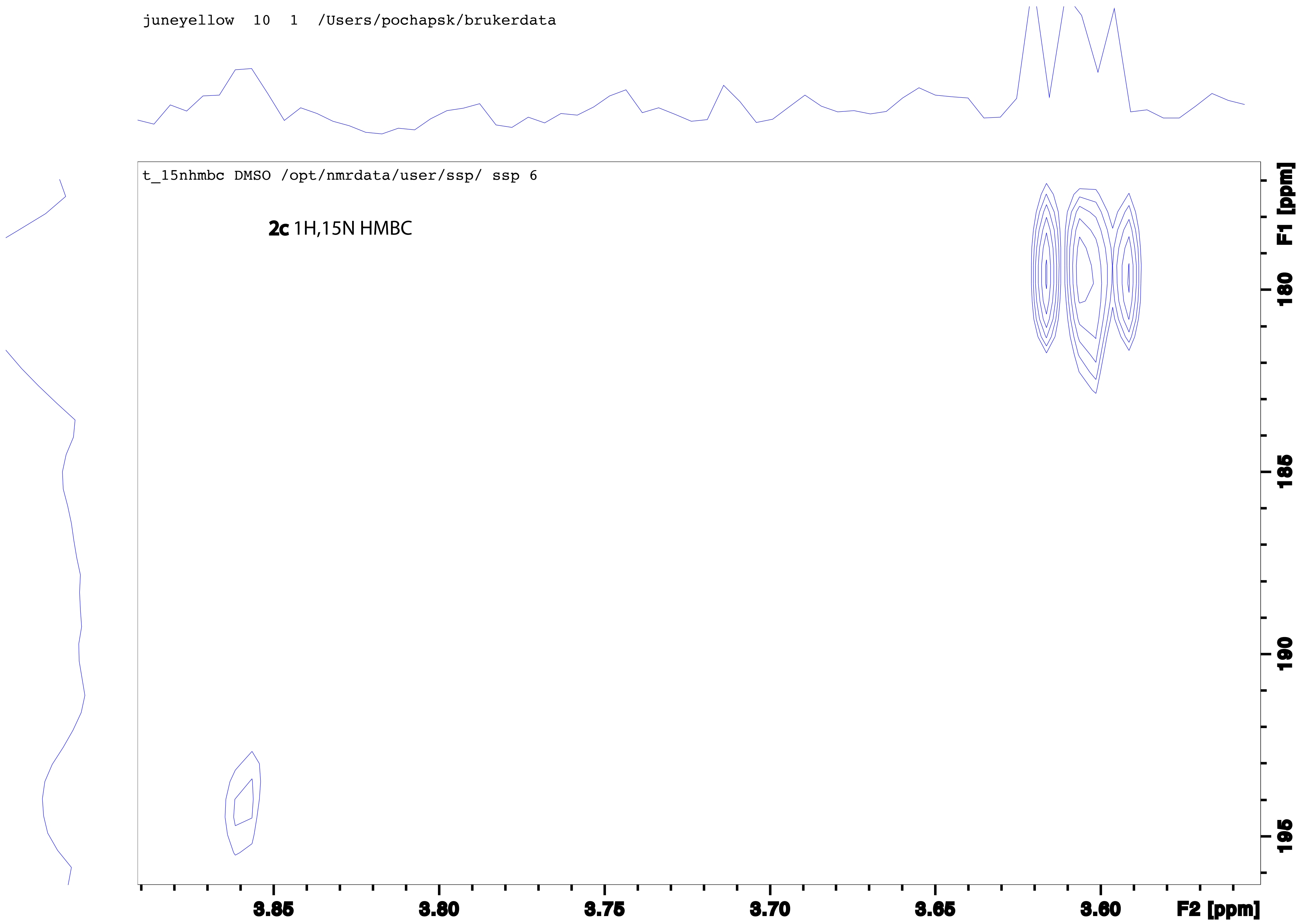
juneyellow 10 1 /Users/pochapsk/brukerdata
t_15nhmbc DMSO /opt/nmrdata/user/ssp/ ssp 6
2c 1H,15N HMBC
F1 [ppm]
180
185
190
195
3.85
3.80
3.75
3.70
3.65
3.60
F2 [ppm]

junegreen 1 1 /Users/pochapsk/brukerdata

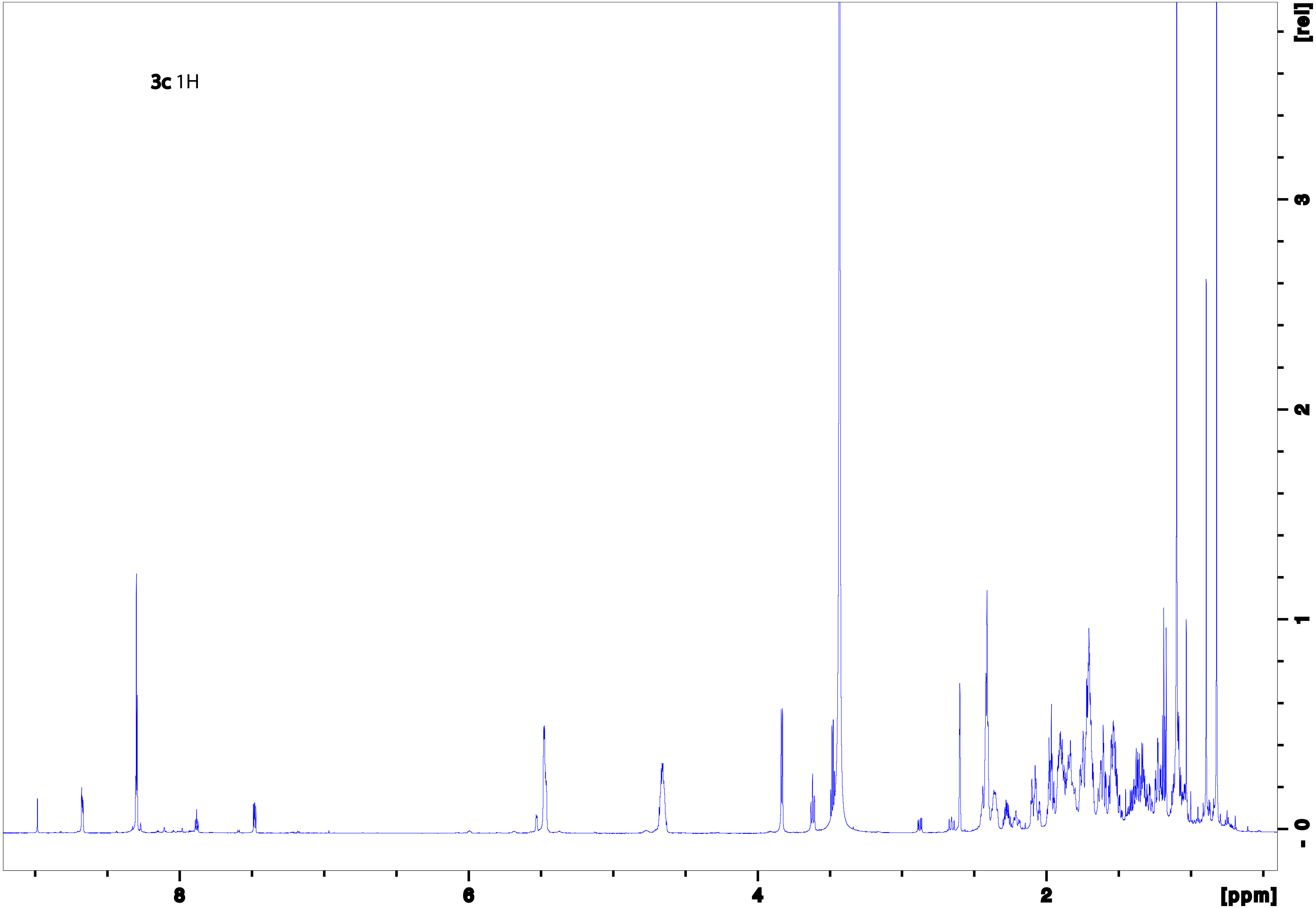

junegreen 2 1 /Users/pochapsk/brukerdata

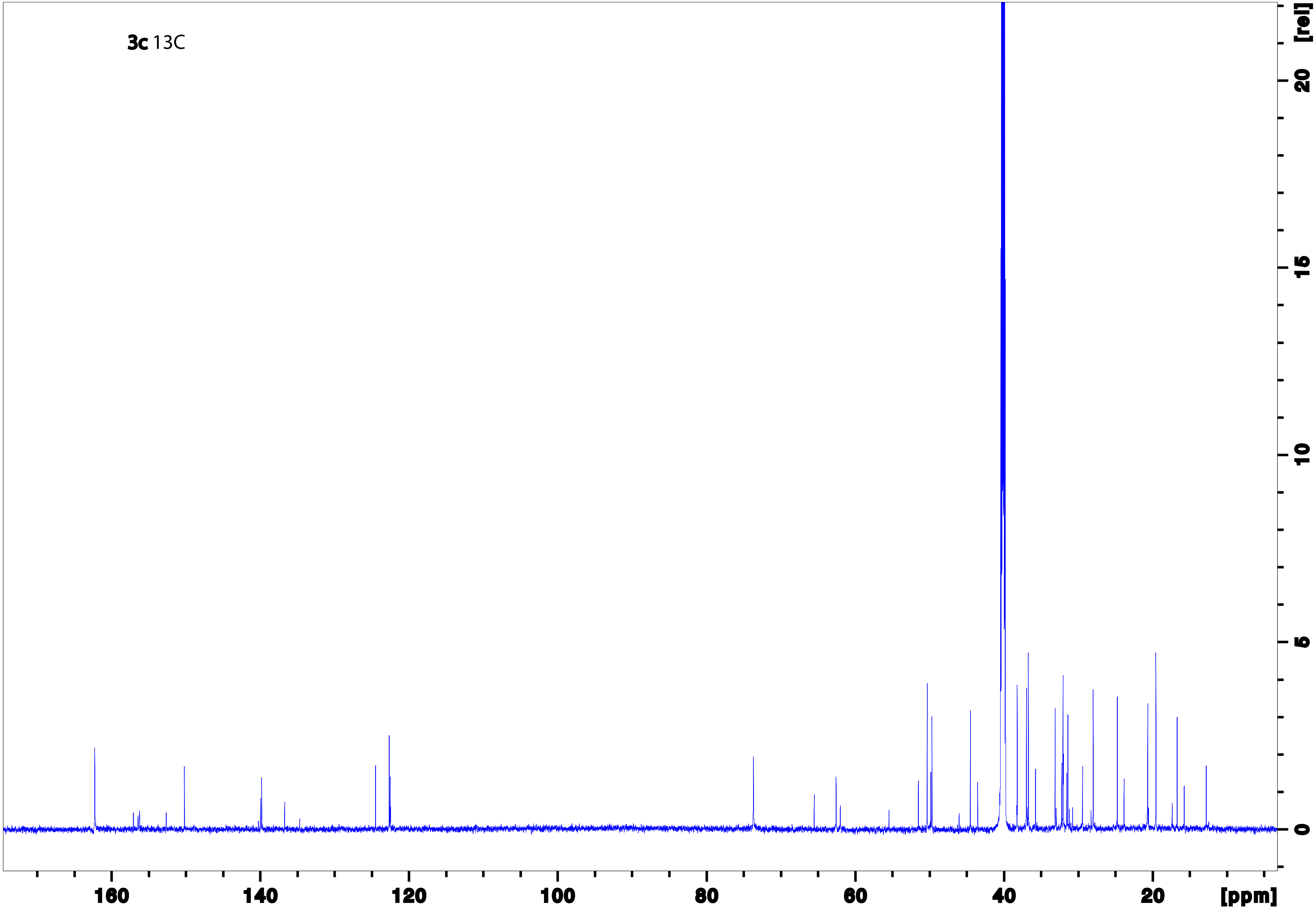

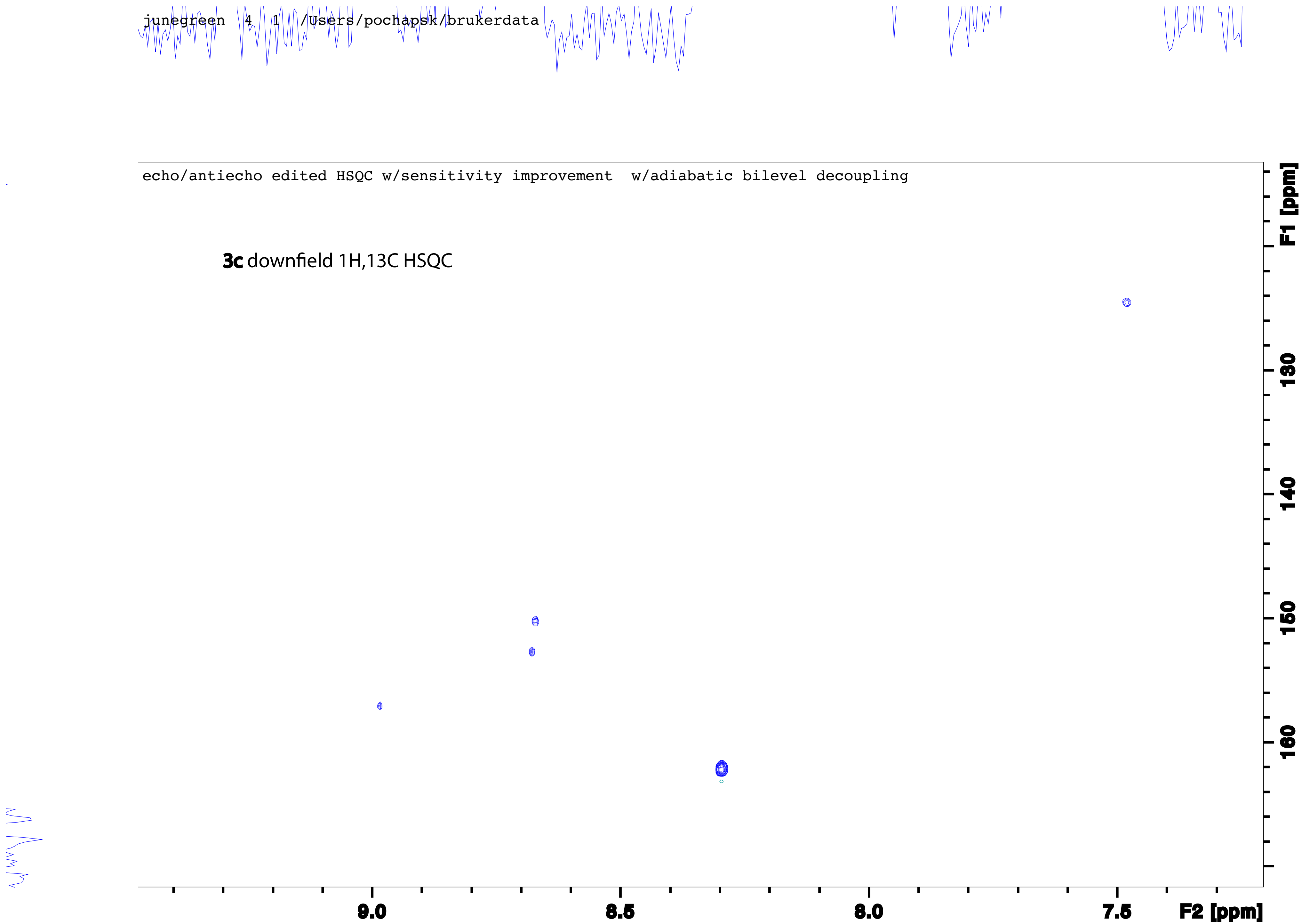
junegreen 4 1 /Users/pochapsk/brukerdata
echo/antiecho edited HSQC w/sensitivity improvement w/adiabatic bilevel decoupling
3c downfield 1H,13C HSQC
130
140
150
160
F1 [ppm]
9.0
8.5
8.0
7.5
F2 [ppm]

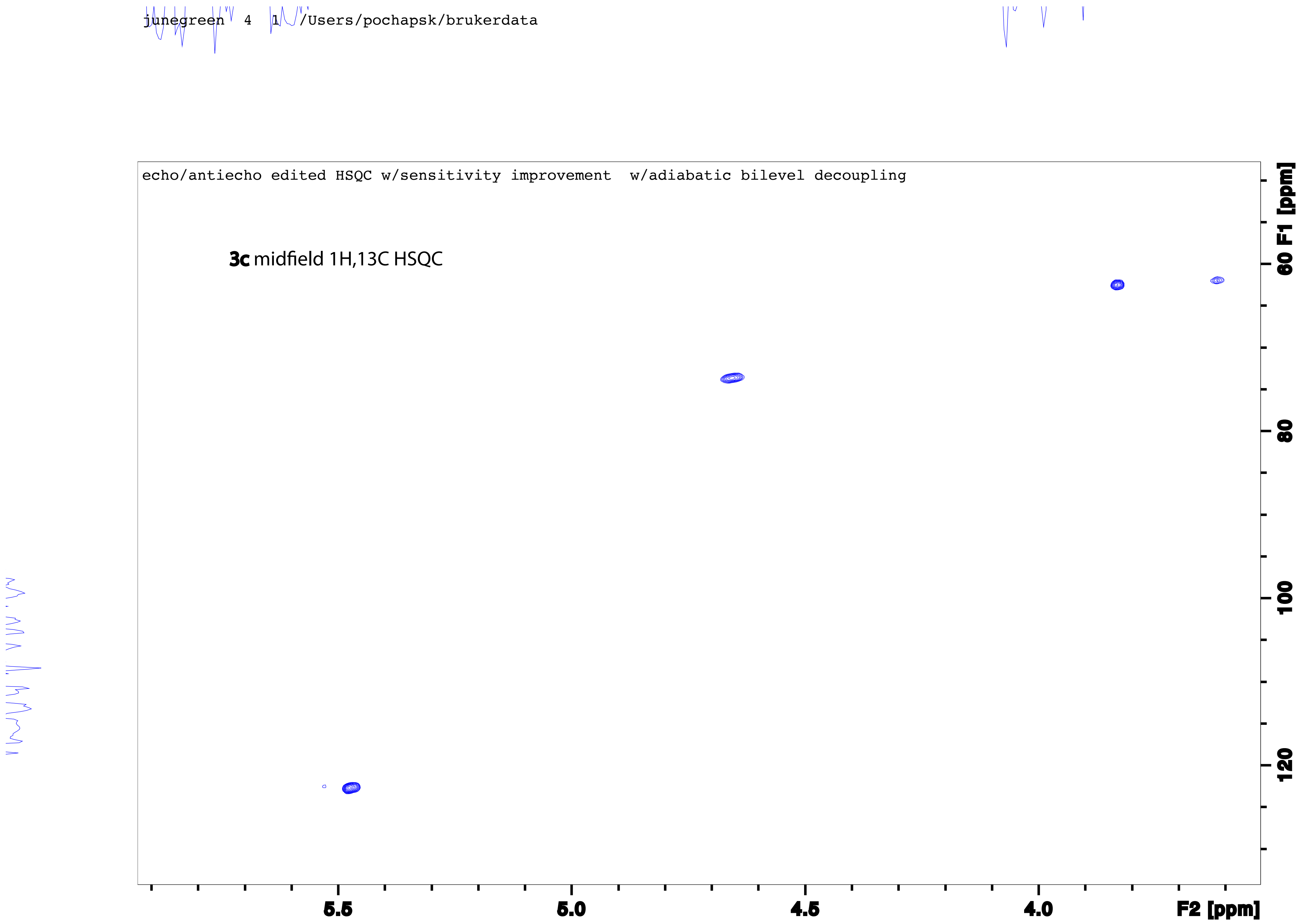

junegreen 4 1 /Users/pochapsk/brukerdata
echo/antiecho edited HSQC w/sensitivity improvement w/adiabatic bilevel decoupling
3c midfield 1H,13C HSQC
60
80
100
120
F1 [ppm]
5.5
5.0
4.5
4.0
F2 [ppm]

junegreen 4 1 /Users/pochapsk/brukerdata

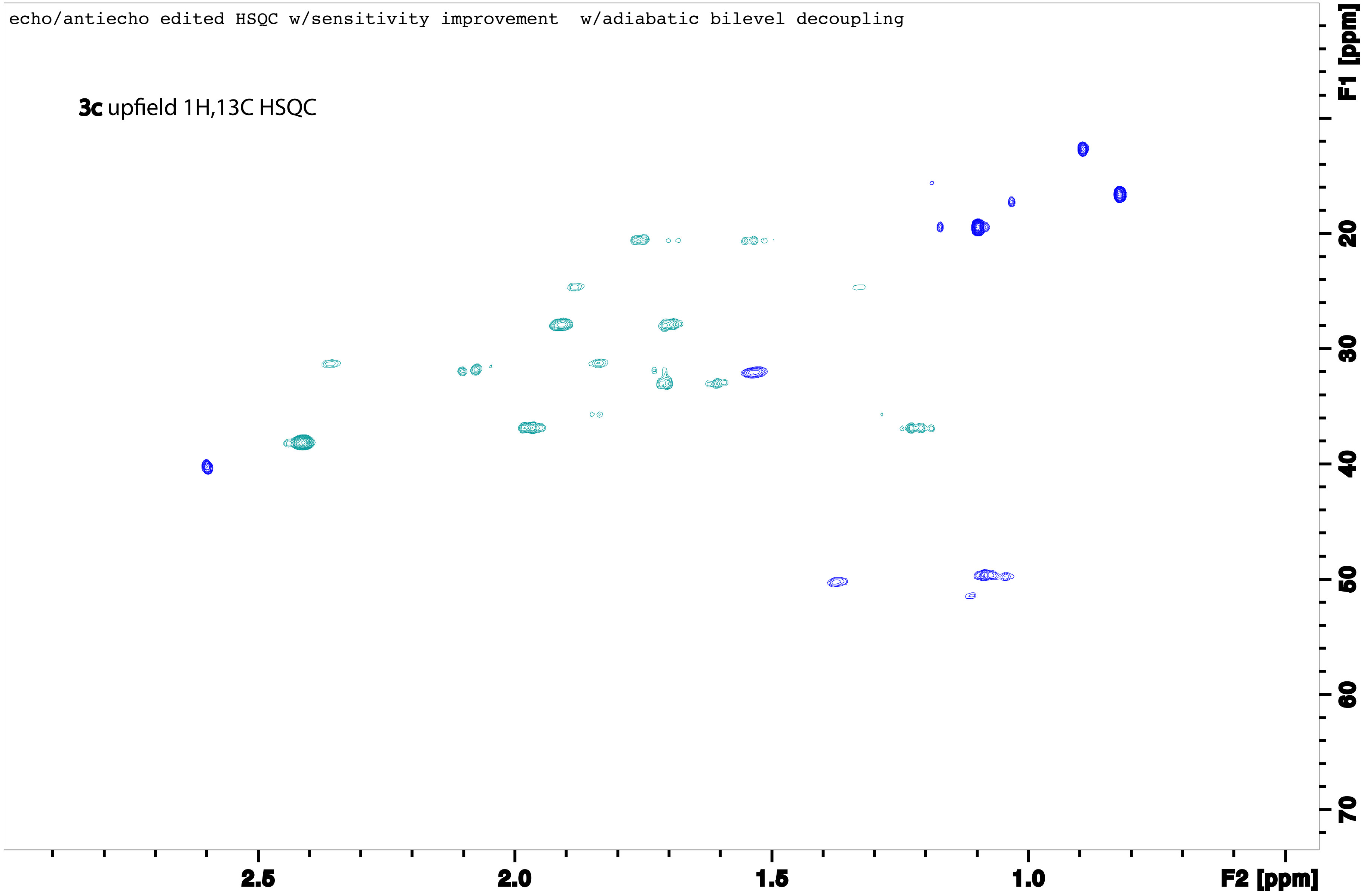

junegreen 5 1 /Users/pochapsk/brukerdata

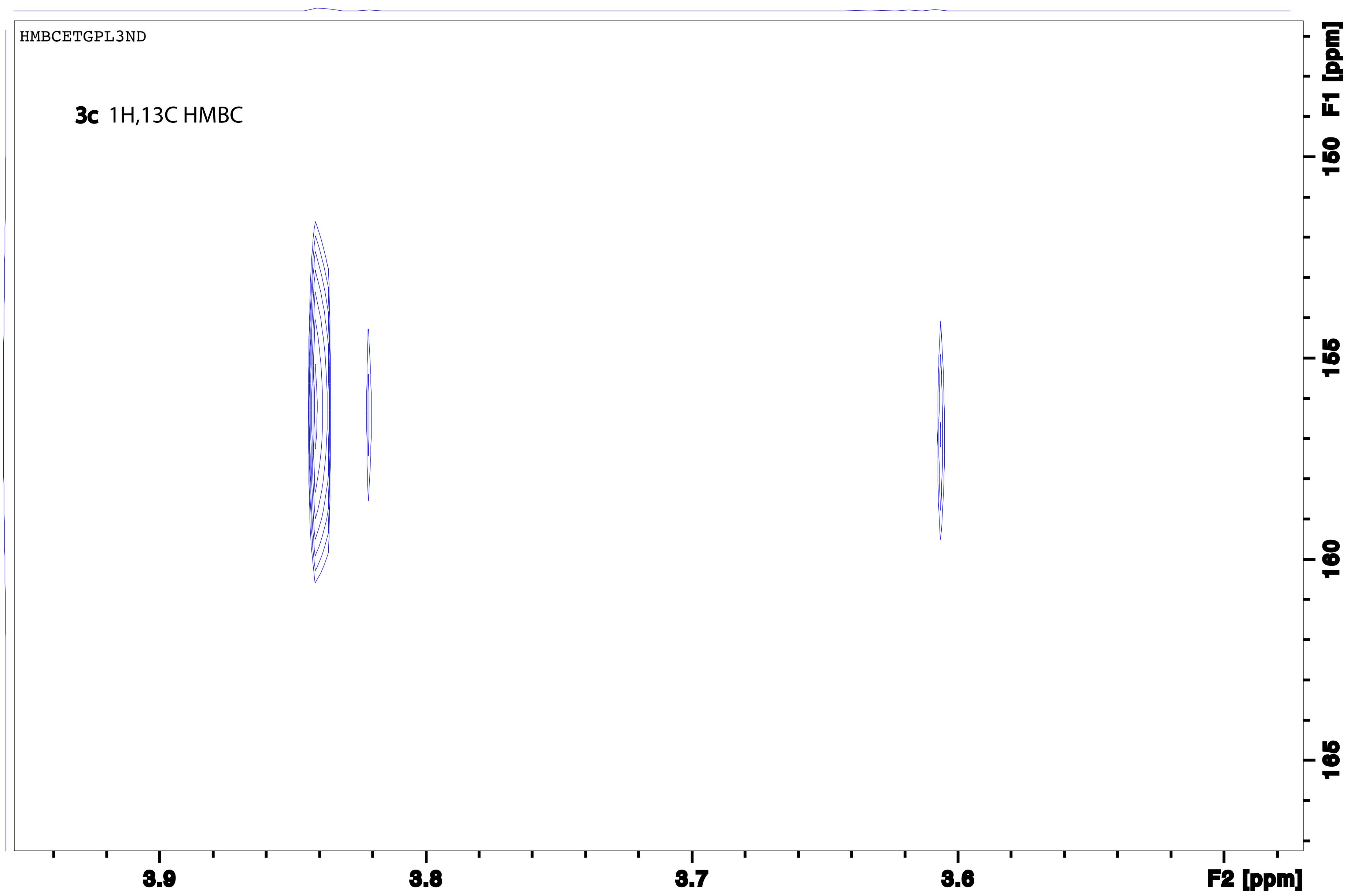

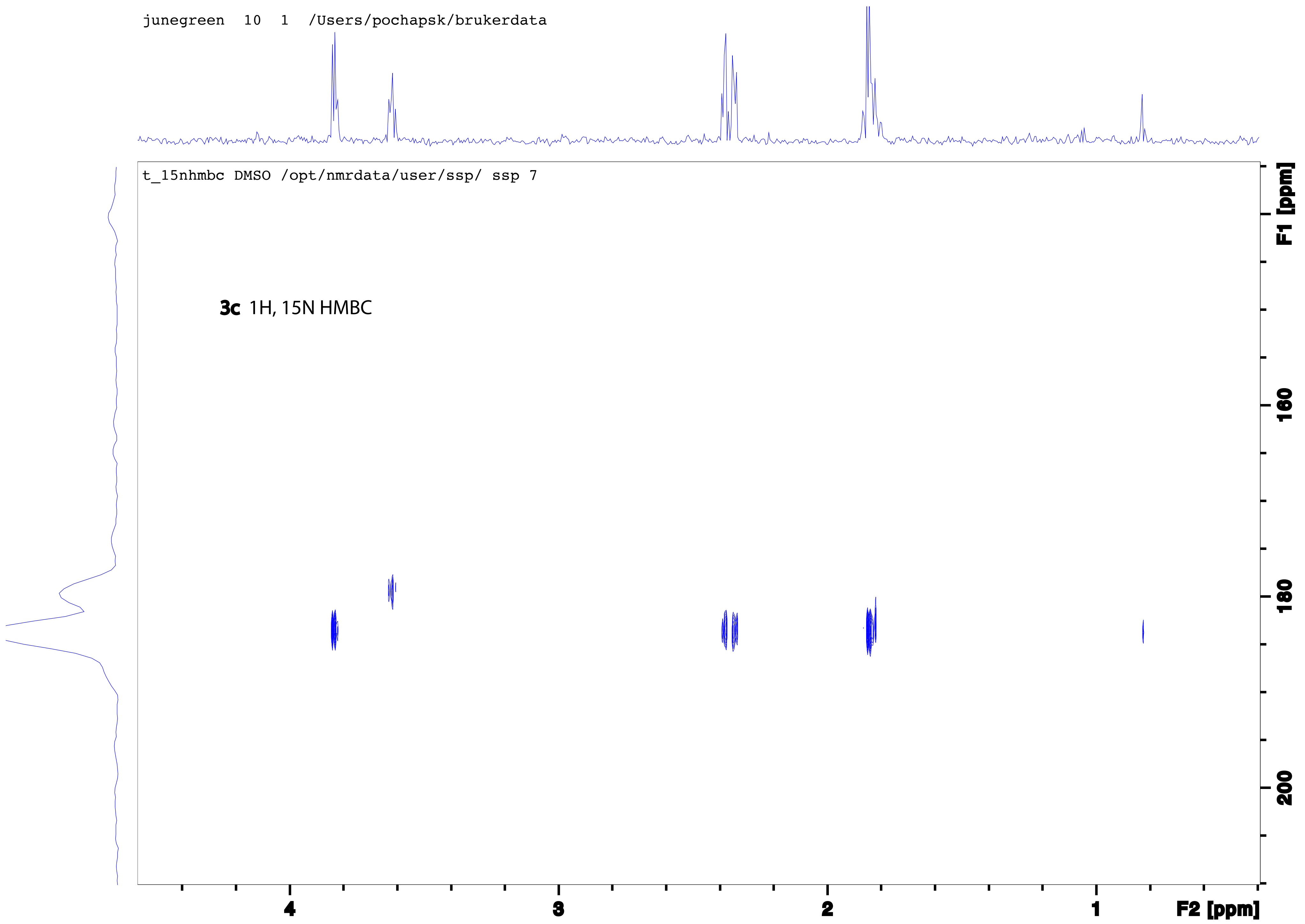
junegreen 10 1 /Users/pochapsk/brukerdata
t_15nhmbc DMSO /opt/nmrdata/user/ssp/ ssp 7
3c 1H, 15N HMBC
F1 [ppm]
160
180
200
4
3
2
1
F2 [ppm]

junered 1 1 /Users/pochapsk/brukerdata

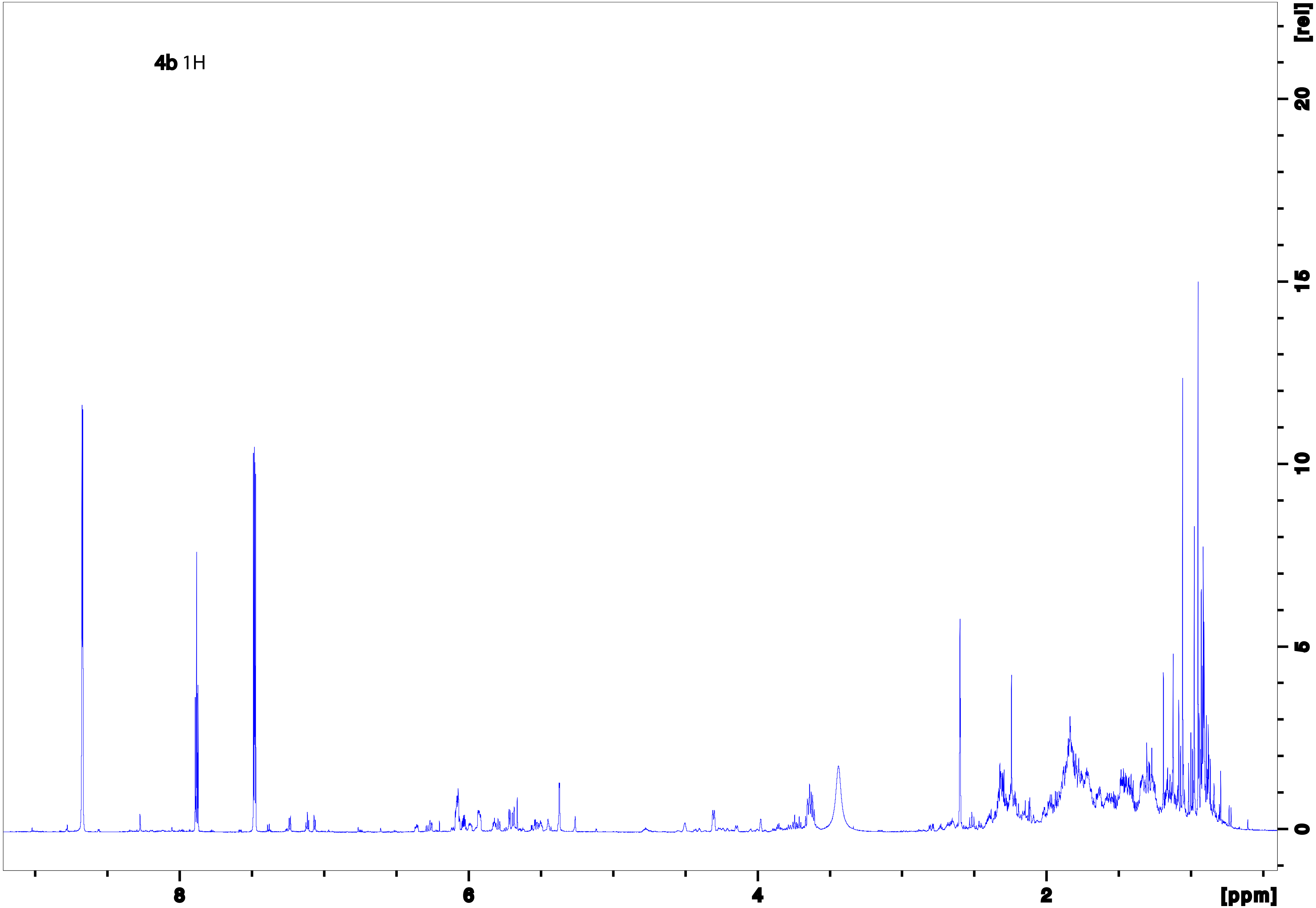

junered 2 1 /Users/pochapsk/brukerdata

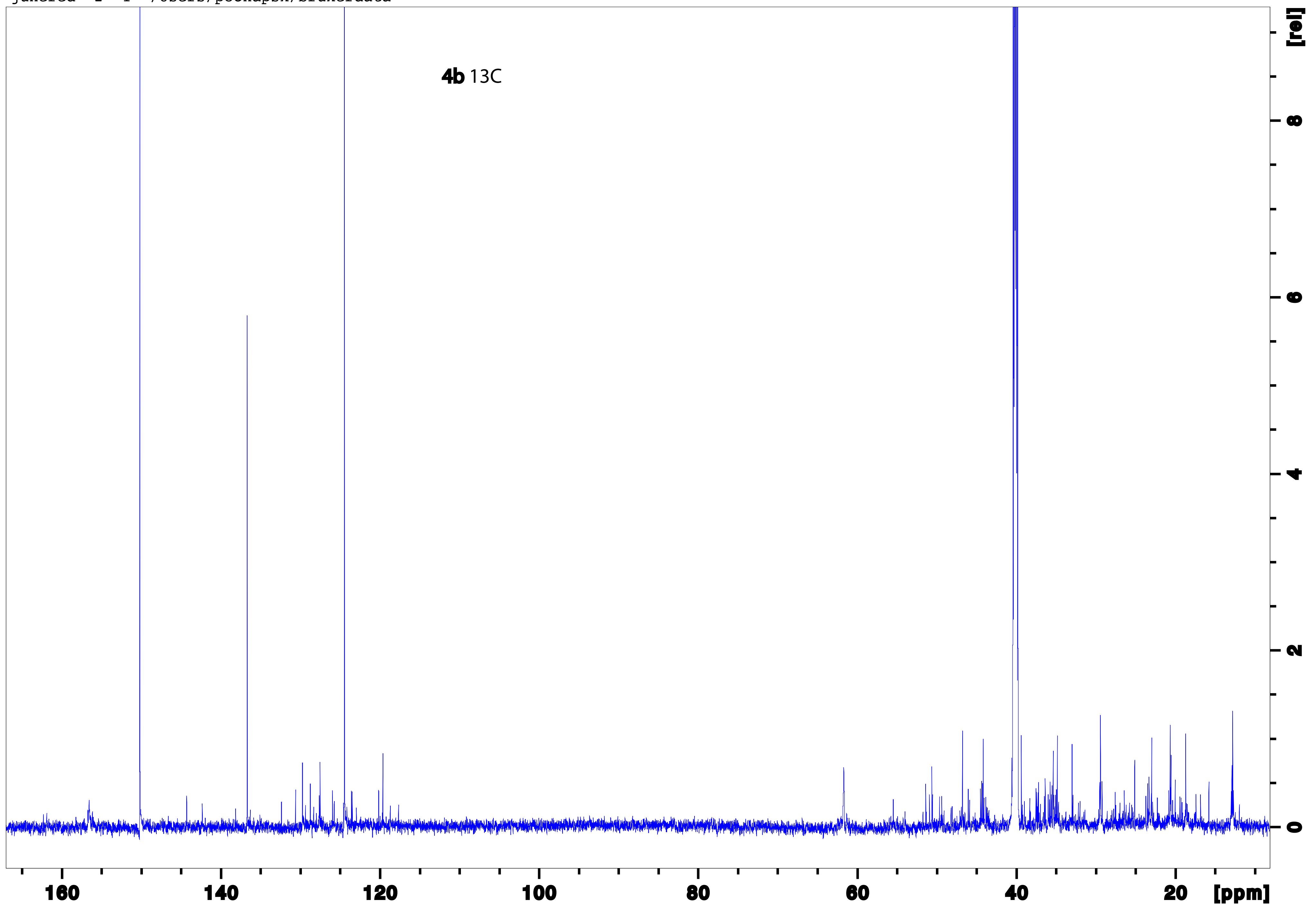

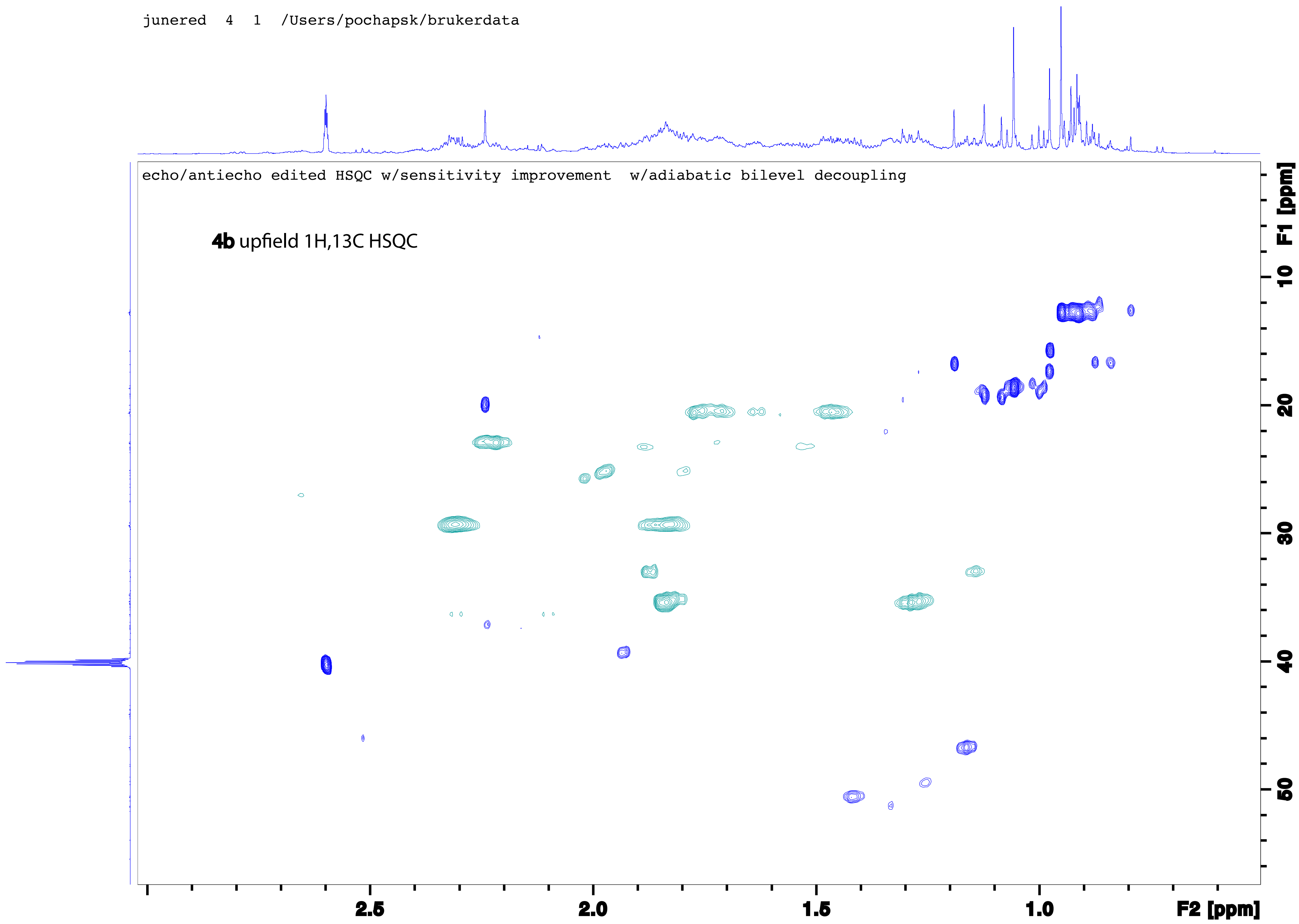
junered 4 1 /Users/pochapsk/brukerdata
echo/antiecho edited HSQC w/sensitivity improvement w/adiabatic bilevel decoupling
4b upfield 1H,13C HSQC
10
20
30
40
50
F1 [ppm]
2.5
2.0
1.5
1.0
F2 [ppm]

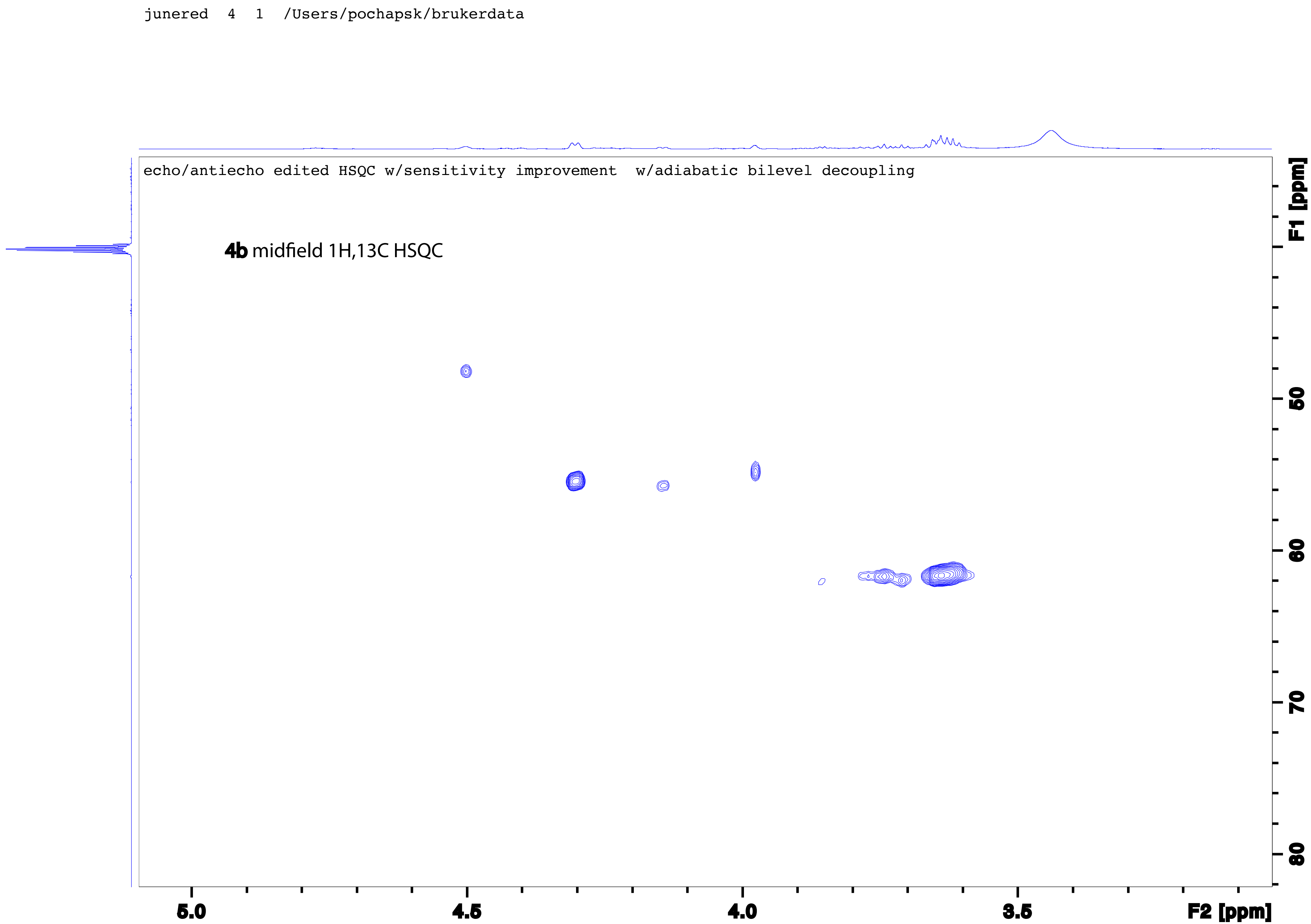
junered 4 1 /Users/pochapsk/brukerdata
echo/antiecho edited HSQC w/sensitivity improvement w/adiabatic bilevel decoupling
4b midfield 1H,13C HSQC
F1 [ppm]
50
60
70
80
5.0
4.5
4.0
3.5
F2 [ppm]

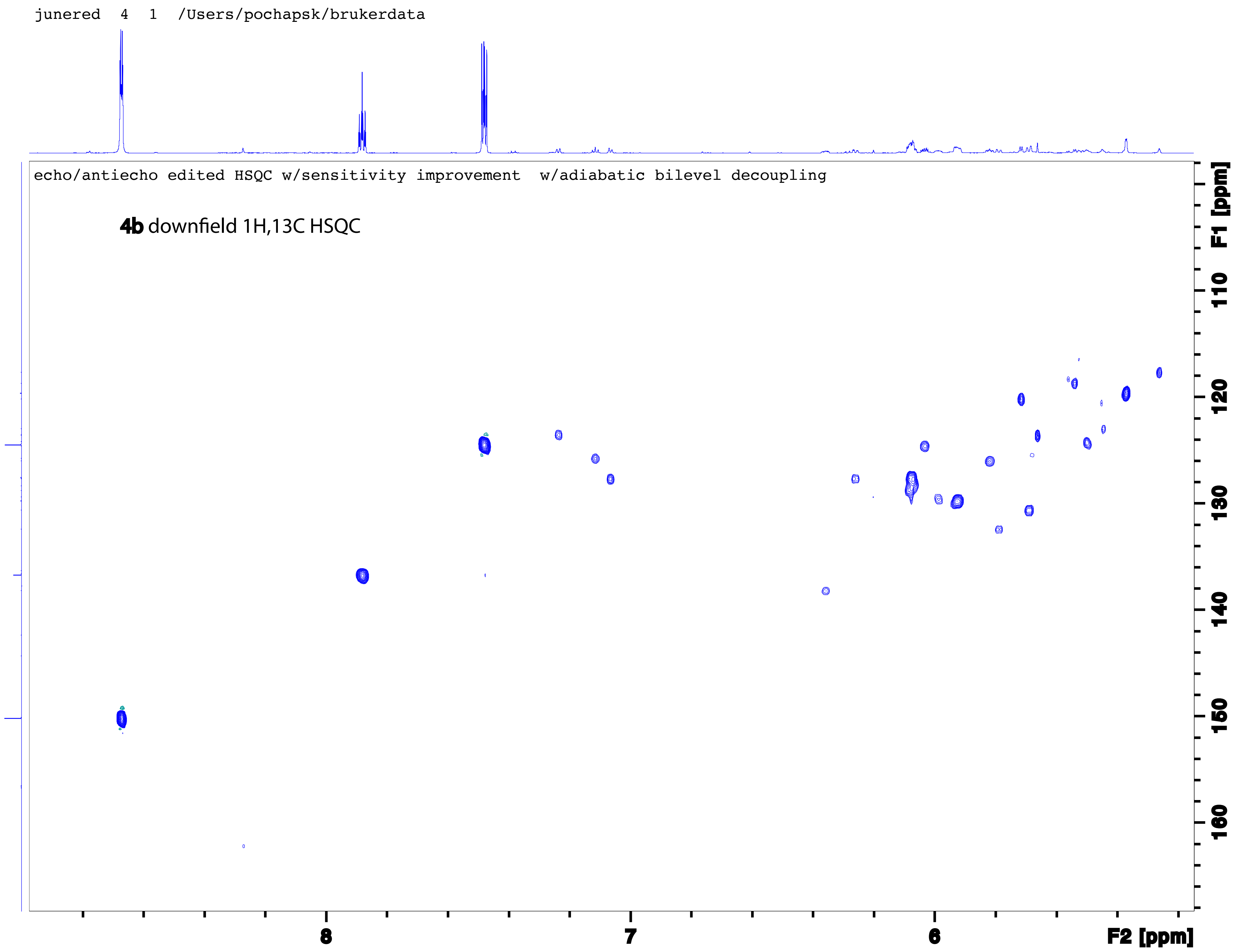
junered 4 1 /Users/pochapsk/brukerdata
echo/antiecho edited HSQC w/sensitivity improvement w/adiabatic bilevel decoupling
4b downfield 1H,13C HSQC
F1 [ppm]
110
120
130
140
150
160
8
7
6
F2 [ppm]

junered 5 1 /Users/pochapsk/brukerdata

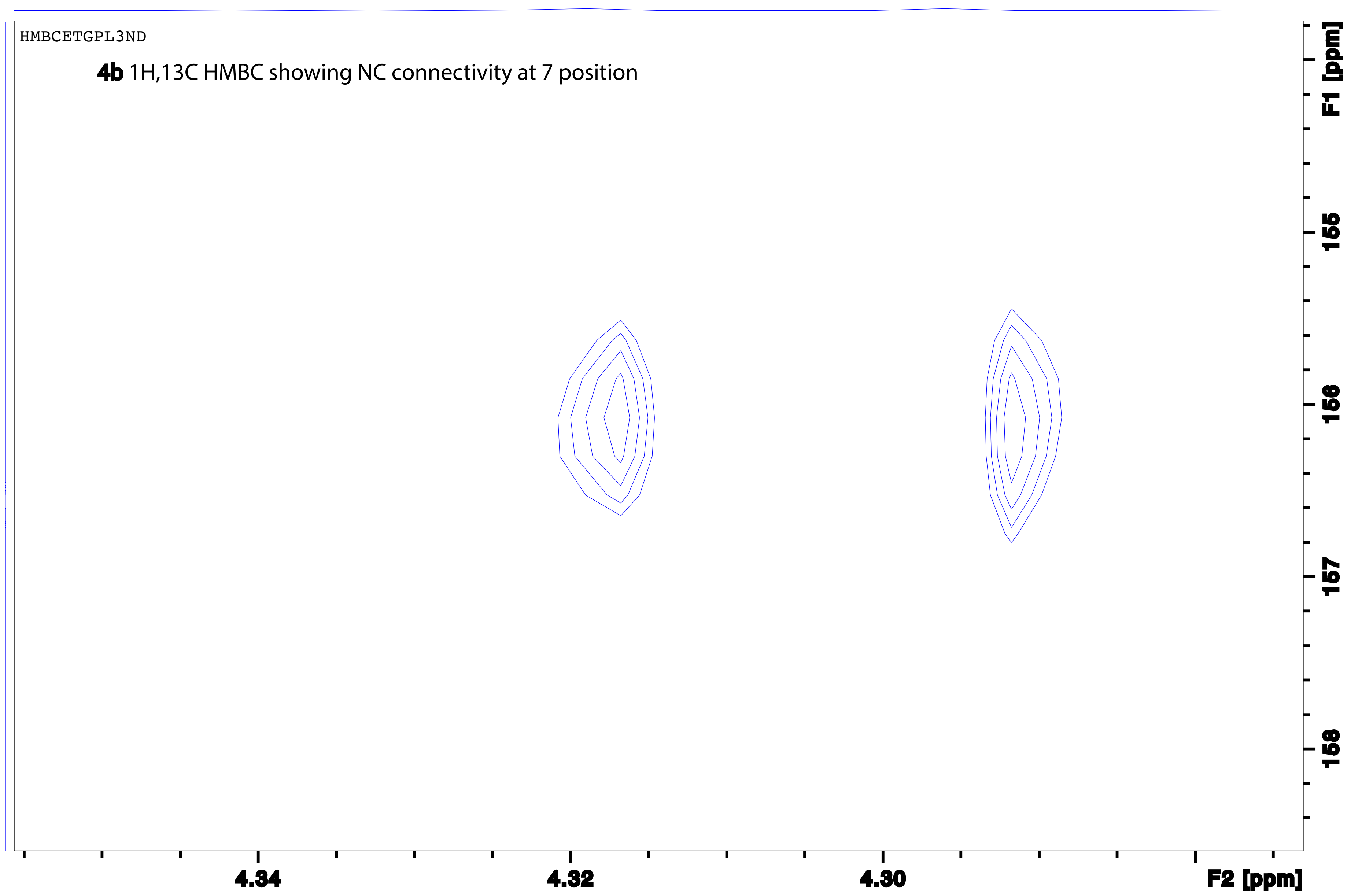

4b 1H,13C HMBC showing NC connectivity at 7 position

junered 5 1 /Users/pochapsk/brukerdata

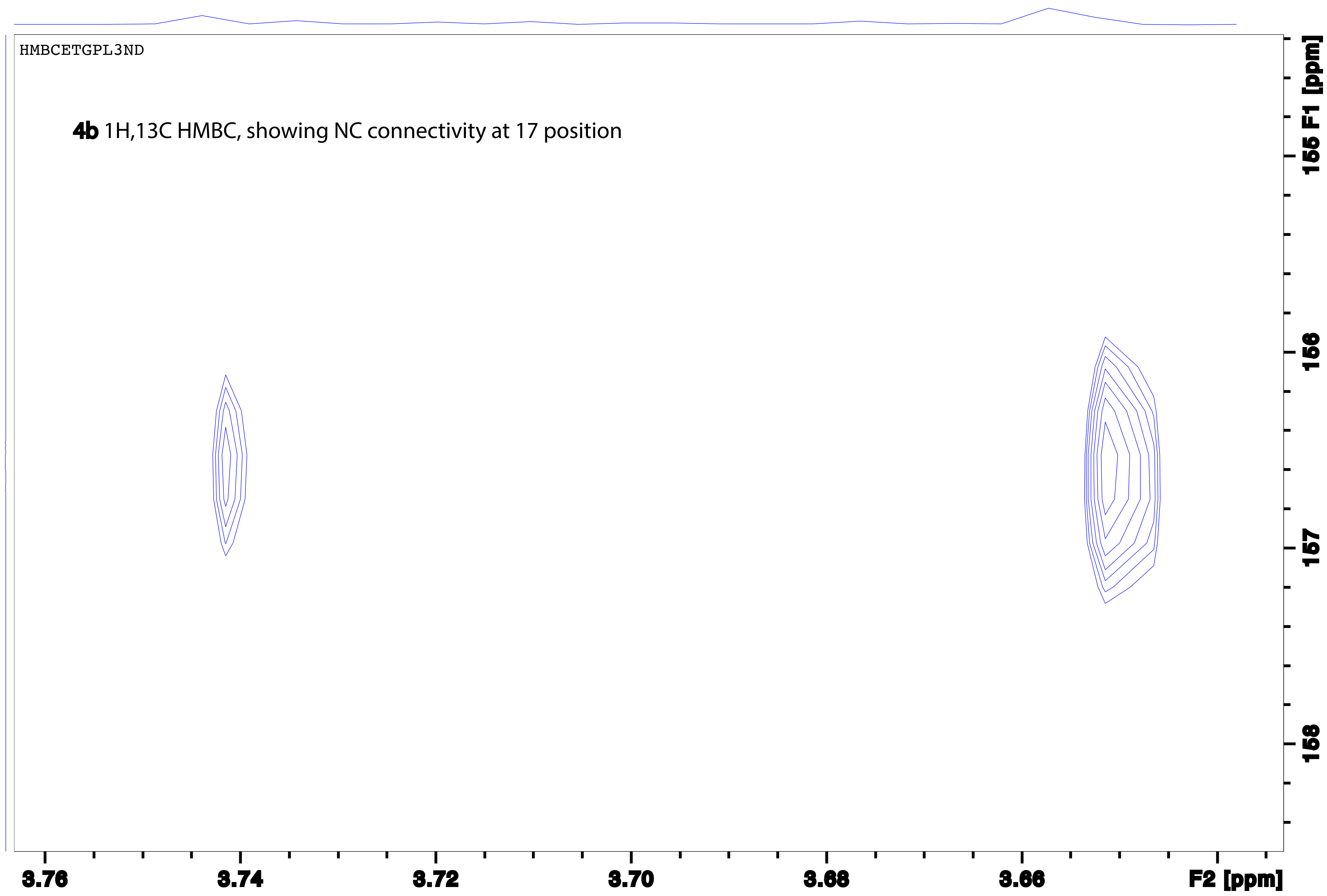

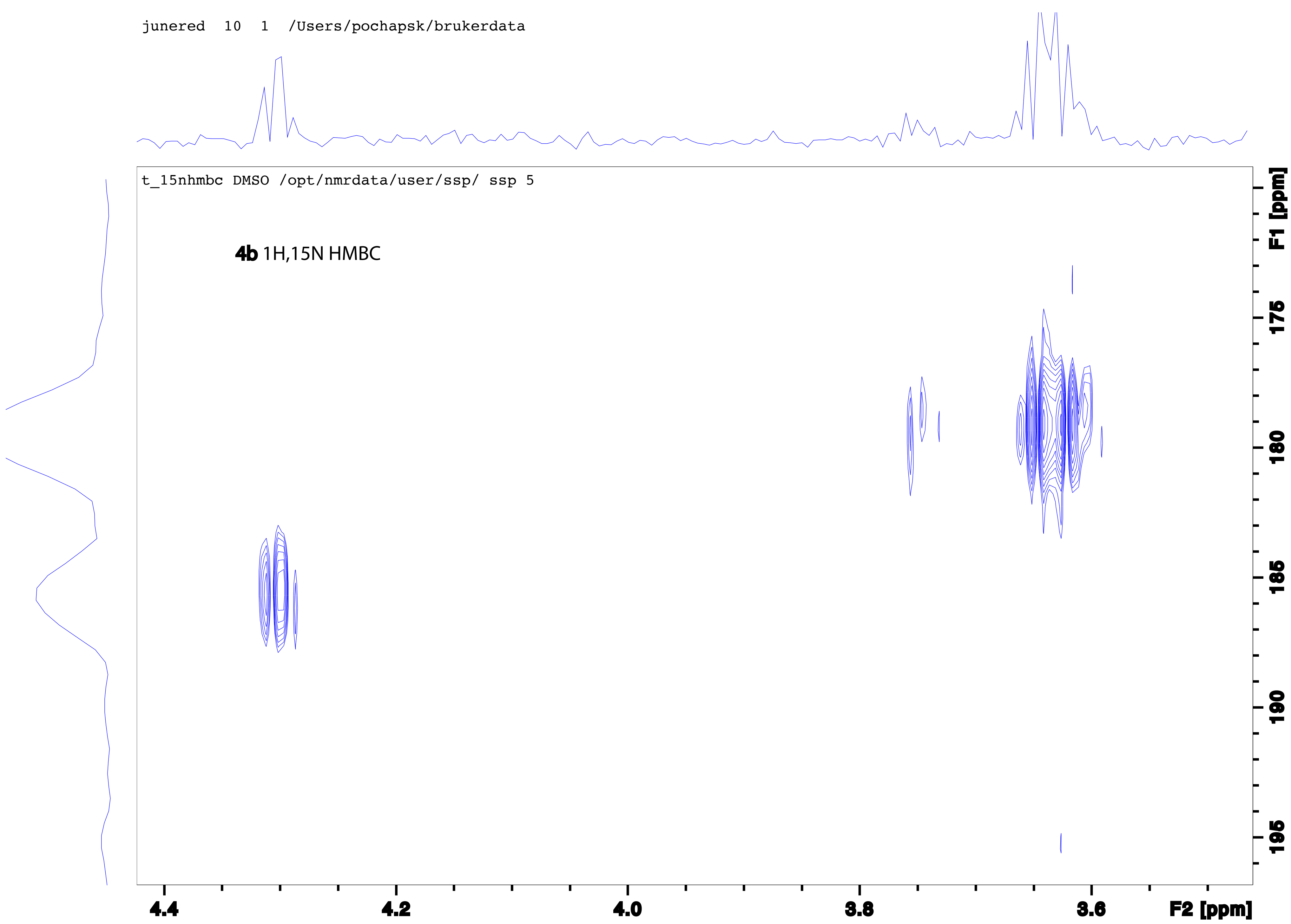
junered 10 1 /Users/pochapsk/brukerdata
t_15nhmbc DMSO /opt/nmrdata/user/ssp/ ssp 5
4b 1H,15N HMBC
175
180
185
190
195
F1 [ppm]
4.4
4.2
4.0
3.8
3.6
F2 [ppm]
